# “Reliable organisms from unreliable components” revisited: the linear drift, linear infinitesimal variance model of decision making

**DOI:** 10.3758/s13423-022-02237-3

**Published:** 2023-01-31

**Authors:** Philip L. Smith

**Affiliations:** Melbourne School of Psychological Sciences, The University of Melbourne, Vic., Melbourne, 3010 Australia

**Keywords:** Decision making, Diffusion model, Accumulator model, Sequential-sampling model

## Abstract

Diffusion models of decision making, in which successive samples of noisy evidence are accumulated to decision criteria, provide a theoretical solution to von Neumann’s (1956) problem of how to increase the reliability of neural computation in the presence of noise. I introduce and evaluate a new neurally-inspired dual diffusion model, the linear drift, linear infinitesimal variance (LDLIV) model, which embodies three features often thought to characterize neural mechanisms of decision making. The accumulating evidence is intrinsically positively-valued, saturates at high intensities, and is accumulated for each alternative separately. I present explicit integral-equation predictions for the response time distribution and choice probabilities for the LDLIV model and compare its performance on two benchmark sets of data to three other models: the standard diffusion model and two dual diffusion model composed of racing Wiener processes, one between absorbing and reflecting boundaries and one with absorbing boundaries only. The LDLIV model and the standard diffusion model performed similarly to one another, although the standard diffusion model is more parsimonious, and both performed appreciably better than the other two dual diffusion models. I argue that accumulation of noisy evidence by a diffusion process and drift rate variability are both expressions of how the cognitive system solves von Neumann’s problem, by aggregating noisy representations over time and over elements of a neural population. I also argue that models that do not solve von Neumann’s problem do not address the main theoretical question that historically motivated research in this area.

In 1956, the great applied mathematician John von Neumann published an article based on a set of lectures given at Caltech entitled “Probabilistic logics and the synthesis of reliable organisms from unreliable components” (Von Neumann, 1956). In it, he considered the effects of probabilistic variation on the fidelity with which neurons in the brain and central nervous system perform the computational task of translating perception into action. Von Neumann identified such variation, which he associated with errors in information transmission in the central nervous system, as the principal obstacle to reliable biological computation. His solution to the reliability problem was aggregation or multiplexing: A signal carried by a single neuron might be in error, but a signal carried by a bundle of neurons was more likely to be correct than not, and the bulk of his article focused on how to characterize and bound the error in automata composed of bundles of binary Pitts-McCulloch neurons (McCulloch & Pitts, 1943).

Von Neumann conceptualized neural computation in terms of elementary logic functions rather than as operations on analogue representations, but the theoretical problem he identified and his proposed solution to it remain at the heart of contemporary cognitive psychology and the way we theorize about cognitive processes and try to model them. My aim in this article is to present a new diffusion model of two-choice decision making, the *linear-drift, linear infinitesimal variance* (LDLIV) model, which I argue provides a more complete and satisfying solution to von Neumann’s problem than do previous models of this kind. The model has many feature in common with other diffusion decision models but its particular theoretical interest is that its architecture and process assumptions correspond more closely to what we would expect from a cognitive process synthesized from and implemented by elementary neural units.

I have used von Neumann’s problem as a focal point for this article because my larger theoretical aim, along with presenting and evaluating the new model, is to distinguish models that seek to provide a solution to von Neumann’s problem from those that do not. Most of the published work on decision models during the past few decades has focused on questions of comparative model fit and predictive adequacy rather than on the more fundamental theoretical questions of exactly what a model explains and how it explains it. By “how a model explains something” I mean the process assumptions or explanatory constructs from which its predictions are derived. In the “Discussion” section I argue that a model’s ability to provide a theoretical solution to von Neumann’s problem should be *the* primary criterion for judging a model’s adequacy. I have sought to situate the new model in this larger context because I believe the theoretical content of a model is at least as important as its ability to fit data and my aim is to try to restore questions of the former kind to the center of the debate.

## Cognition as probabilistic neural computation

The idea of probabilistic variation in cognitive representations discussed by von Neumann was not a new one, but was discovered in psychology nearly 100 years earlier by Fechner, who found there was trial-to-trial variability in whether pairs of similar stimuli were judged to be the same or different (Fechner, 1860). The work of understanding the implications of Fechner’s discovery continued into the twentieth century and led to Thurstone’s law of comparative judgment (Thurstone, 1927) and signal detection theory (Green & Swets, 1966). But even before these developments, Fechner in 1860 already had a well-developed theory of decision making based on Gauss’s 1809 theory of errors (Sheynin, 1979), which substantially foreshadowed the modern theory (Link, 1994; Wixted, 2020). In Gauss’s theory, a measurement based on an aggregation of elements, each independently subject to error, will be normally distributed. Fechner attributed the variability in same-different decisions to aggregated measurement errors of this kind. His insight was thus much as the same as von Neumann’s 100 years later: Variability in cognitive representations is a reflection of aggregation over similar elements that are independently subject to error. It follows that reliability can be improved by increasing the number of elements that compose the representation, as von Neumann proposed. The idea that cognitive representations are normally distributed, which Link (1994) attributes to Fechner, was carried over into the law of comparative judgment and signal detection theory (Luce, 1994), both of which, in their most common forms, assume normally-distributed representations.

As important as signal detection theory is to mainstream psychology, there is an important dimension missing from its account, which is time. If we assume, following Fechner and von Neumann, that variability in cognitive representations reflects noise or error in the neural elements that compose them, then we would expect this variability to be present not only on the time scale of behavioral decisions but on the time scale of neural events as well — that is, from moment to moment as well as from decision to decision. Long before it was possible to record decision-related neural activity from single cells in awake behaving monkeys, multiple-look psychophysical experiments performed in the early days of signal detection theory showed that discrimination accuracy improved in proportion to the square root of the number of times a brief stimulus was presented (Swets, Shipley, McKey, & Green, 1959). A square-root law improvement is predicted if the decision is based on the sum of successive, independent, noisy representations of the stimulus. That this is so suggests the brain solves von Neumann’s problem in time in the same way as it solves it in space: by aggregation. Von Neumann’s solution — which is also the statistician’s solution — is to improve reliability by aggregating across noisy elements that code the same sensory quantity. The results of Swets et al. further suggest that (a) those elements are noisy or variable in time, and (b) the brain improves reliability by aggregating, or summing noisy representations over time.

These ideas were developed systematically during the 1960s and 1970s in psychology in the class of *sequential-sampling* decision models by authors such as Stone (1960), La Berge (1962), Audley & Pike (1965), Edwards (1965), Laming(1968), Link & Heath (1975), Vickers (1970, 1979), abd Ratcliff (1978). These models were inspired, in part, by the sequential statistics of Abraham Wald (1947) and, while they differed from one another in points of detail (Ratcliff and Smith, 2004; Smith & Ratcliff, 2015), they shared the common assumptions that evidence entering the decision process is perturbed by noise or error on a shorter time scale than that of the individual decision and that the decision is made by aggregating or accumulating independent samples of evidence over time.

## Neurally-principled models of decision making

The classical sequential-sampling models were derived from theoretical principles and the best of them successfully accounted for choice probabilities (accuracy) and response times (RT) from behavioral experiments before it was possible to make recordings of decision-related neural activity in awake, behaving animals. When such recordings became possible technically (Roitman & Shadlen, 2002; Thompson, Hanes, Bichot, & Schall, 1996), the picture they provided was in striking agreement with the account proposed by the models. Recordings from the oculomotor cortex of monkeys performing eye-movement decision tasks typically show: (a) moment-to-moment statistical variation, reminiscent of von Neumann’s “unreliable components;” (b) a progressive increase in firing rates with the time elapsing from stimulus onset, and (c) responses that are time-locked to the point at which the firing rate reaches a threshold or criterion level (Hanes & Schall, 1996). Hanes and Schall argued that these properties are consistent with the idea that the neurons implement a process of evidence accumulation like that proposed by sequential-sampling models. In the intervening years the picture has become increasingly clear and has provided further support for their original insight (Forstmann, Ratcliff, & Wagenmakers, 2016; Smith & Ratcliff, 2004).

As researchers have continued to probe the relationship between decision processes and their neural implementation, there has been a corresponding impetus to develop models that are more neurally realistic or more deeply grounded in neurocomputational principles. “Neural realism” is, of course, a relative term, as neural processes can be modeled at different levels of resolution and what is realistic at one level is an oversimplification at another (Gerstner & Kistler, 2002; Tuckwell, 1988), but, in practice, questions of neural realism have tended to focus either on the decision *architecture* or the decision *process*. By “architecture” I mean the assumptions made about how evidence for different decision alternatives is represented. By “process” I mean the stochastic process that is used to represent the accumulating evidence. The debate about architecture has mainly been about whether the evidence for different alternatives is represented by a single stochastic process or whether each alternative is represented by its own evidence state or process. The debate about process has been about whether evidence is accumulated in discrete time or continuously and whether it is continuously distributed or comes in discrete chunks (Ratcliff & Smith, 2004; Smith & Ratcliff, 2015). Most neurocomputational studies have assumed diffusion processes of some kind — that is, continuous-time, continuous-state Markov processes (Rogers & Williams, 2000) – and I likewise focus on diffusion models.

The emphasis on diffusion processes is a natural one theoretically, because diffusion representations can be derived from either bottom-up or top-down approaches to characterizing evidence accumulation neurocomputationally. Examples of the bottom-up approach are the Poisson shot-noise models of Smith (2010) and Smith and McKenzie (2011), in which a diffusion process is obtained as the limit of processes that represent the flux in postsynaptic potentials induced by a sequence of action potentials. Examples of the top-down approach are the diffusion approximations to the mean-field dynamics of the Ising decision maker of Verdonck and Tuerlinckx (2014) and the spiking neuron model of Wang (2001, 2002), and Wong & Wang (2006). The Ising decision maker is a recurrent network of Pitts-McCulloch neurons like those analyzed by Von Neumann (1956) whereas the Wang model is a recurrent network of spiking neurons. In both models the decision process is represented as a process of energy minimization in which the stable, or attractor, states of the system correspond to decision states. The properties of recurrent models can be characterized using mean-field approximations, in which the average firing rate of neurons in a network is derived from an analysis of its global dynamics (Gerstner and Kistler, 2002). Verdonck and Tuerlinckx (2014) and Roxin and Ledberg (2008) derived diffusion approximations to the mean-field equations for the Ising decision maker and the Wang model, respectively, using central-limit theorem arguments, which assumed the number of participating neurons is large (Fechner’s assumption).

To date, the most widely and successfully applied model of two-choice decisions is the diffusion decision model (Ratcliff, 1978; Ratcliff & McKoon, 2008), which represents evidence accumulation as a Wiener, or Brownian motion, process. The Wiener process is the unique member of the class of diffusion processes that is also an independent-increments process, that is, in which each new increment to the process is statistically independent of those preceding it. One of the most straightforward ways to derive the Wiener process mathematically is as the continuous-time limit of a simple random walk (Feller, 1968; Ratcliff, 1978), which is a Markov process in which the increments to the process are either + 1 or − 1. Neurcomputationally, the simple random-walk can be thought of as arising from the difference of two processes, each composed of binary Pitts-McCulloch neurons like those analyzed by Von Neumann (1956) and assumed by Verdonck and Tuerlinckx (2014), which individually are either firing (+ 1) or silent (0) at any instant. The two component processes are assumed to represent the evidence for the two decision alternatives. As a model of decision making, the Wiener process has the attractive property that it implements a continuous-time version of Wald’s (1947) sequential probability ratio test (Bogacz, Brown, Moehlis, Holmes, & Cohen, 2006), which guarantees a form of statistical optimality in homogeneous environments, in which decisions are all of the same level of difficulty.

Despite the success of the diffusion model it has sometimes been seen as lacking neural realism. Its derivation, as the limit of a simple random walk implemented by populations of Pitts-McCulloch neurons, can be criticized on the grounds that such neurons radically simplify the properties of real neurons, although this has not commonly been identified as a serious problem. Indeed, most other models that propose diffusion process representations of evidence accumulation have made similar simplifying assumptions (Roxin & Ledberg, 2008; Verdonck & Tuerlinckx, 2014). In contrast, the shot-noise models of Smith (2010) and Smith and McKenzie (2011), although they also abstract away from the properties of real neurons, derive diffusion process representations from the flux of postsynaptic potentials induced by a train of action potentials. Neurally-motivated critiques of the diffusion model have tended to focus either on the model architecture or the use of the Wiener process — as opposed to some other process — to represent evidence accumulation. Three features of the neural processes involved in decision making not represented in the diffusion model have led to the claim it lacks neural realism:

1. Neural firing rates typically increase in cells responding to both selected and unselected decision alternatives, suggesting that evidence for competing alternatives is represented anatomically by separate processes rather than by a single process.
2. Neural firing rates saturate at high stimulus intensities, so any diffusion process model of evidence accumulation represented by firing rates should similarly be bounded.
3. Neural firing rates are never negative, so any diffusion process model of the evidence represented by them should similarly be nonnegative.

Some researchers have investigated alternatives to the single-process Wiener model that align more closely with these principles, although it remains the benchmark model that any alternative model must equal in order to be credible and, indeed, the importance of the principles themselves can be debated (see the “Discussion” section). Gold and Shadlen (2001), for example, in defense of single-process models, argued that a log-likelihood-ratio evidence accumulator can be obtained by taking the difference between members of pairs of mirror-image units they called “neurons and antineurons,” whose activities individually are nonnegative. That being so, the requirement that neural evidence accumulation processes must be nonnegative arguably loses some of its force. However, the anatomical locus for the hypothetical signed difference process has not been identified and it is also the case that evidence for competing decision alternatives is represented separately as far downstream as the superior colliculus, which is the last neural structure before the response in eye-movement decision tasks. The existence of these anatomically late representations adds to the plausibility of the separate accumulators account.

Three main alternatives to the process and architecture assumptions of the diffusion model have been investigated:

1. **Independent accumulators.** Several researchers have investigated models in which evidence for competing alternatives is modeled by racing diffusion processes (Logan, Van Zandt, Verbruggen, & Wagenmakers, 2014; Ratcliff & Smith, 2004; Ratcliff, Hasegawa, Hasegawa, Smith, & Segraves, 2007; Smith & Ratcliff, 2009; Tillman, Van Zandt, & Logan, 2020). Ratcliff et al., (2007) and Smith and Ratcliff (2009) called these models *dual diffusion* models, which is the terminology I adopt here. Other accumulator model architectures, like the simple accumulator or recruitment model (Audley & Pike, 1965; La Berge, 1962), the Poisson counter model (Smith & Van Zandt, 2000; Townsend & Ashby, 1983), and the Vickers accumulator (Smith & Vickers, 1988; Vickers, 1970; 1979), give poorer RT distribution predictions than models based on racing diffusion processes (Ratcliff & Smith, 2004). Usher and McClelland (2001) proposed a model with mutual inhibition between the accumulators on grounds of neural plausibility and some researchers have reported that these kinds of models give a better fit to data (Ditterich, 2006; Kirkpatrick, Turner, & Sederberg, 2021). Others have found that models with independent accumulators give good accounts of data (Ratcliff & Smith, 2004; Smith & Ratcliff, 2009; Tillman et al., 2020) including neural data. Ratcliff et al., (2007) found that a dual-diffusion model predicted choice probabilities, RT distributions, and neural firing rates in the superior colliculi of monkeys performing an eye-movement decision task. There was no evidence of suppression in the firing rates in cells associated with the non-chosen alternative at the time of the response, as predicted by mutual-inhibition models. The advantage of models with independent accumulators is that they are tractable analytically whereas models with interactive accumulators are not.
2. **Bounded evidence accumulation.** Instead of modeling evidence accumulation using the Wiener process, some researchers have modeled it using the Ornstein-Uhlehbeck (OU) process, which is a diffusion process with leakage or decay (Busemeyer and Townsend, 1992; 1993; Diederich, 1995; Smith, 1995; Smith & Ratcliff, 2009; Smith, Ratcliff, & Sewell, 2014; Usher & McClelland, 2001). Unlike the Wiener process, the OU process has a stationary distribution At long times, evidence in the OU process has a Gaussian distribution with constant mean and variance (Karlin & Taylor, 1981; Smith, 2000). The stationarity property is a diffusion counterpart of the saturation property of neural firing rates. Although the OU process has sometimes been proposed on grounds of neural realism, whether it improves a model’s ability to account for data depends on its other assumptions. Ratcliff and Smith (2004) found that an OU process in a two-boundary, single-process architecture performed no better than a Wiener process and when the OU decay parameter was free to vary it went to zero, which is the Wiener process. However, Smith and Ratcliff (2009) found that in a dual-diffusion architecture a process with decay, that is, an OU process, performed better.
3. **Reflecting boundaries.** The state, or evidence, space of the Wiener and OU processes is the entire real line, $\mathbb {R}$, which is inconsistent with the use of these processes to model neural firing rates, which can never be negative. The simplest way to constrain a process to the positive half line, $\mathbb {R}^{+}$, is with a *reflecting* boundary. When a process reaches such a boundary it is reflected back into the state space and continues accumulating (Karlin & Taylor, 1981). Models with reflecting boundaries have been proposed by Diederich (1995), Smith and Ratcliff (2009), and Usher and McClelland (2001).^1^ Apart from considerations of neural plausibility, in the Usher and McClelland model the reflecting boundary is needed to stabilize its dynamics. Without it, if the evidence in one of the accumulators goes negative then the mutual inhibition between them becomes mutual excitation and the model becomes unstable.

## The linear drift, linear infinitesimal variance model

**Fig. 1:**
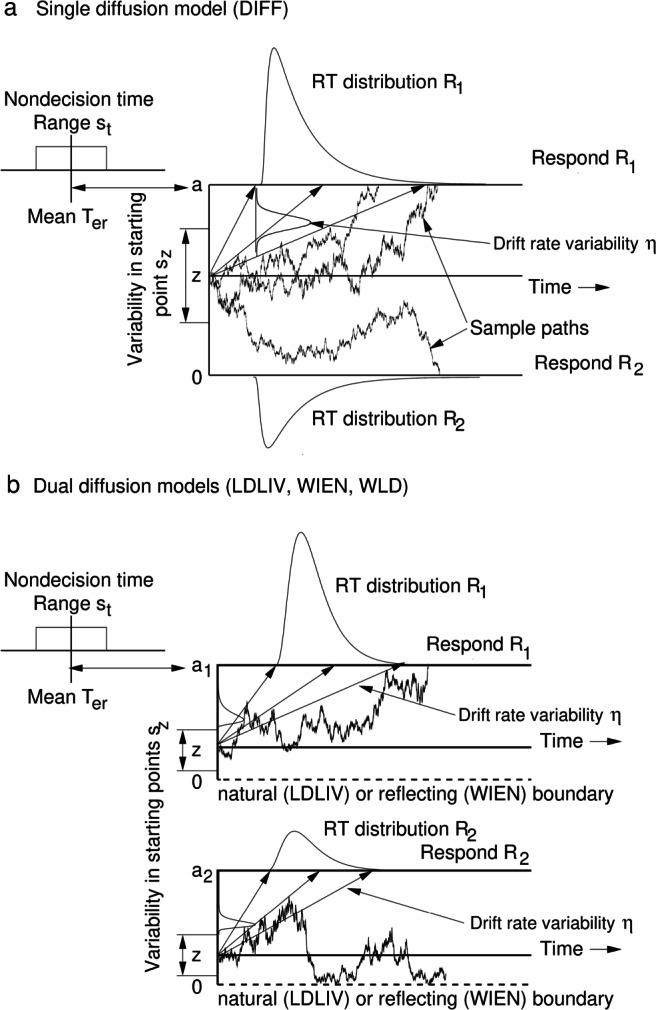
Sequential-sampling decision models. (a) Single-process Wiener diffusion model (DIFF). Starting at *z* the process accumulates evidence between decision criteria at 0 and *a*. There is variability in the drift rate, *ξ*, with standard deviation *η*, starting point, *z*, with range *s*_*z*_, and nondecision time, *T*_er_, with range *s*_*t*_. (b) Dual diffusion models. Evidence is accumulated by racing diffusion processes starting at *z* with decision criteria at *a*_1_ and *a*_2_. In the linear drift, linear infinitesimal variance model (LDLIV), zero is a natural boundary that constrains the processes to the positive real line $\mathbb {R}^{+}$, and evidence accumulates with rate *q* and decays with rate − *p*. In the racing, reflecting Wiener model (WIEN) the processes are constrained below by reflecting boundaries at *r* and evidence accumulates with rate *μ* with no decay. The racing Walds model (WLD) is unbounded below. The LDLIV and WIEN models have across-trial variability in drift rate, starting point, and nondecision time; the WLD model has only across-trial variability in starting point and nondecision time

In this article, I present and evaluate a new diffusion model, the LDLIV model, which embodies the three principles above. The model is shown in Fig. 1, along with the standard diffusion model and two related models that are evaluated below, to which it may be contrasted. Specifically, the LDLIV model assumes that: (a) evidence for competing alternatives is accumulated by independent processes; (b) the processes are bounded; and (c) the processes are nonnegative. An attractive feature of the LDLIV process that recommends it as a model of decision-making is that its dynamics naturally constrain it to the positive half line, $\mathbb {R}^{+}$, without artificial or externally-imposed bounds. This contrasts with the dual diffusion models of Ratcliff et al., (2007) and Smith and Ratcliff (2009) and the leaky competing accumulator model of Usher and McClelland (2001) and the model of simple RT of Diederich (1995), in which the evidence processes are constrained by reflecting boundaries. Although the reflecting boundary endows these models with the right properties mathematically, it can be objected that the boundary is not a part of the process itself and lacks any clear neural interpretation. In contrast, the LDLIV process lives naturally, in the mathematical sense of the word, on $\mathbb {R}^{+}$.

Another attractive property of the LDLIV process is that it becomes progressively more noisy, or variable, as the activation state increases. This property captures the general tendency for neural firing rates to become more variable as their means increase (Rieke et al., 1997). Model neurons typically embody this property as a basic design feature (Gerstner and Kistler, 2002, p. 177, Tuckwell, 1988, p. 197). The Wiener process in the standard diffusion model becomes more variable with the passage of time, but the change in variability (diffusion rate) is independent of the change in the mean (drift rate). In contrast, in the LDLIV process, the change in variability depends on the current state. This property seems to better capture the dynamics of noisy evidence growth in neural populations than does the assumption that drift and diffusion rates are independent of each other. A third attractive feature of the LDLIV model is that the distribution of evidence states in the process is positively skewed, as we would expect a diffusion approximation to neural firing rate distributions to be. This contrasts to the Wiener and OU processes, both of which predict normally distributed evidence states. The theoretical interest of the LDLIV process for decision modelers comes from the fact that it embodies the three design principles stated above and also that it is possible to derive explicit mathematical predictions for it using integral equation methods similar to those described by Smith (2000) and Smith and Ratcliff (2022). The methodological contribution of this article is to introduce an explicit integral equation representation of the first-passage time distribution for the LDLIV process and to use it to fit data.

I compare the LDLIV model to two dual-diffusion models that relax one or more of the three principles. One is a model composed of racing Wiener diffusion processes between absorbing and reflecting boundaries. This model embodies Principles 1 and 3 but relaxes Principle 2 (boundedness). I also obtain explicit mathematical predictions for this model using integral equations. The other model, which also relaxes Principle 3 (positivity), is composed of racing unconstrained Wiener diffusion processes. This model is attractive because of its conceptual and mathematical simplicity and was recently advocated as an alternative to the standard diffusion model by Tillman et al., (2020). I compare these models based on independent racing processes to the standard diffusion model.

Mathematically, a diffusion process is defined by specifying a pair of functions or coefficients: the *drift rate* and the *diffusion rate* (or diffusion coefficient), which are referred to as the *infinitesimal moments* of the process. The drift rate characterizes the expected change in the process in a unit interval and the diffusion rate characterizes the change in its variance (Bhattacharya and Waymire 1990, Ch. 7; Cox & Miller 1965, Ch. 5; Karlin & Taylor^1981^, Ch. 15). The square root of the diffusion rate is the *infinitesimal standard deviation*. In the most general diffusion process, the drift and diffusion rate may depend both on time, *t*, and the position of the process in the evidence space, *x*. We can write a stochastic differential equation (SDE) for such a process, *X*_*t*_, which specifies the random change in the process during a small time interval, *dt*, as follows

1 $$ dX_{t} = A(X_{t}, t) dt + \sqrt{B(X_{t}, t)} dW_{t}, $$

where *A*(*x*, *t*) and *B*(*x*, *t*) are, respectively, the drift and diffusion rates, and *dW*_*t*_ is a zero-mean Gaussian increment whose standard deviation in a small interval of duration Δ*t* is of the order $\sqrt {\Delta t}$. An alternative way to characterize such processes is via partial differential equations (the so-called Kolmogorov backward and forward equations).^2^ For first-passage time problems, which characterize the time required to pass through an absorbing boundary, and which arise in relation to RT modeling, the relevant equation is the backward equation (Ratcliff, 1978). The LDLIV process (Feller, 1951) satisfies the SDE

2 $$ dX_{t} = (pX_{t} + q) dt + \sqrt{2\sigma X_{t}} dW_{t}, $$

with drift rate *A*(*x*, *t*) = *px* + *q* and diffusion rate *B*(*x*, *t*) = 2*σx*. The LDLIV process has a linear drift rate, like the OU process. When the state-dependent coefficient of the drift rate, *p*, is negative, it acts as a decay or leakage term, which tends to pull the process back to zero at a rate proportional to its current state, *x*. Like the OU process, leakage bounds the evidence accumulation process, consistent with Principle 2.

Where the LDLIV process differs from the OU process is in the diffusion rate. The OU process has a constant diffusion rate, which means it can take on both positive and negative values, like the Wiener process. In contrast, the diffusion rate of the LDLIV process is proportional to *x*, which constrains the process to the positive real line, consistent with Principle 3.

In the remainder of this article, I first present explicit integral-equation expressions for the response time and accuracy predictions for the LDLIV model and the Wiener model between absorbing and reflecting boundaries. Predictions for the other two models are available in the literature. I then describe applications of the models to data from two experiments. One is from a numerosity discrimination study by Ratcliff (2008), which crossed stimulus discriminability with a speed-accuracy manipulations. The other is from an attention-cuing study using sinusoidal grating stimuli by Smith, Ratcliff, and Wolfgang (2004). These two studies were used recently by Smith and Ratcliff (2022) as benchmarks to compare fixed-boundary, collapsing-boundary, and urgency-gating models and allowed them to distinguish between competing models. Both studies had multiple levels of stimulus discriminability (four in the Ratcliff study and five in the Smith et al. study) crossed with other experimental manipulations that produced different, and highly constrained, patterns of correct and error RTs, which are important in model comparison.

Many recent studies of decision making in psychology have followed single-cell recording studies of motion perception in neuroscience and have investigated decisions about coherent motion in random dot kinematograms (Dutilh et al., 2019). Indeed, the random dot motion task is now sometimes seen as being the prototype of a decision-making task. Smith and Lilburn (2020) questioned this characterization and argued that, in important respects, the task may be quite atypical. They reviewed evidence from psychophysical temporal integration studies suggesting it requires an unusually long period of perceptual integration to form a representation of the motion signal. Consistent with the psychophysics, they found that the RT distributions and choice probabilities in the Dutilh et al. data were better characterized by a model in which the drift rates depended on the time-varying outputs of a linear filter with an integration time of around 400 ms than by a model in which they were constant. The tasks I considered here can both be considered “typical” in that they have previously been well-described by the standard Wiener diffusion model with constant drift and diffusion rates.

## Integral equation methods for diffusion decision models

Mathematically, the predicted choice probabilities and decision times in a diffusion model are obtained by solving a first-passage time problem, which gives the probability distribution of the time when the process first crosses an absorbing boundary, which is identified in the psychological model with the time at which the accumulating evidence reaches a decision criterion. Classically, the solution of the backward equation for the Wiener process leads to a representation of the RT distributions as an infinite series (Ratcliff, 1978) that can be computed efficiently. The availability of an explicit solution, which has been implemented in several third-party software packages (Vandekerckhove & Tuerlinckx, 2008; Voss & Voss, 2007; Wiecki, Sofer, & Frank, 2013), has been important in encouraging the use of the model in basic and applied settings (Ratcliff, Smith, & McKoon, 2015; 2016). For other, more complex, diffusion processes, infinite-series solutions cannot be obtained and other methods must be sought. For models with interacting processes, like the Usher and McClelland (2001) model, Monte Carlo simulation may be the only or the most practicable way to evaluate them (although see Ditterich (2006) for a finite-state Markov chain approach) and considerable effort has recently gone into developing methods for approximating likelihoods and estimating parameters of simulated decision models (e.g., Turner and Sederberg, 2014; Turner & Van Zandt, 2018; Fengler, Govindarajan, Chen, & Frank, 2021). My own bias is towards models whose first-passage time distributions can be expressed in a mathematically explicit form, because I believe they provide the sharpest theoretical insights into the properties of the underlying processes, and I have accordingly focused my attention on models of this kind.

The integral-equation method was pioneered by Durbin (1971), who used it to compute the power of the Kolmogorov-Smirnov test. The method was subsequently developed by Ricciardi and colleagues (Buonocore et al., 1987; Buonocore, Giorno, Nobile, & Ricciardi, 1990; Ricciardi, 1976; Ricciardi & Sato, 1983)to characterize the firing time distributions of model integrate-and-fire neurons. Early applications to decision processes were described by Heath (1992) and Smith (1995, 1998). A detailed tutorial introduction may be found in Smith (2000) and recent applications to collapsing-boundary and urgency-gating models may be found in Voskuilen, Ratcliff, and Smith (2016) and Smith and Ratcliff (2022). Applications to models with time-varying drift and diffusion rates can be found in Smith and Ratcliff (2009), Smith et al., (2014), and Smith and Lilburn (2020).

For models based on racing diffusion processes, the theoretical quantities of interest are the functions, *g*[*a*(*t*), *t*,|*z*,0], the first-passage time distribution for a diffusion process, *X*_*t*_, starting at *z* at time 0, through an absorbing boundary *a*(*t*) at time *t*, which may be time-varying, as the notation suggests. The integral equation method yields a representation of the first-passage time density function in the form of a Volterra integral equation of the second kind, of the form

3 $$ \begin{array}{@{}rcl@{}} g[a(t), t| z, 0] &=& -2{\Psi}[a(t), t | z, 0] \\&&+ 2 {{\int}_{0}^{t}} g[a(\tau), \tau |  z, 0] {\Psi}[a(t), t| a(\tau), \tau] d\tau. \end{array} $$

In this equation, *g*[*a*(*t*), *t*|*z*,0] is jointly a function of its values at previous times, *τ* < *t*, and of a kernel function, Ψ[*a*(*t*), *t*|*a*(*τ*), *τ*], which depends on the drift and diffusion rates of *X*_*t*_ and which is described in detail below. Equation 3 is derived from a general integral representation of the first-passage time for a diffusion process through an absorbing boundary, attributed to Fortet (1943). A derivation of Eq. 3 based on the Fortet representation is sketched in Appendix A.

A problem with the original Fortet representation, as Durbin (1971) recognized, was that the equation becomes singular as $\tau \rightarrow t $, that is, as the interval of approximation becomes small. This is because the free transition density function of the process, *f*[*a*(*t*), *t*|*a*(*τ*), *τ*], which describes the position of the process in the absence of boundaries, approaches a Dirac delta function, *δ*(*τ* − *t*), which describes a spike of probability mass of infinite amplitude concentrated at the point *t*. This means that simple iterative schemes for evaluating the first-passage time density will become numerically unstable. Durbin considered some ad hoc ways to stabilize the equation but Buonocore et al., (1987) showed that for many processes of interest it is possible to choose the kernel in a way that removes the singularity entirely. With the kernel chosen in this way, the equation may be approximated on a discrete set of points and solved iteratively (Eq. A3). Derivations of the kernel functions for Wiener and OU processes with time-varying drift rates, diffusion rates, and boundaries, with an application to urgency-gating models, can be found in Smith and Ratcliff (2022).

## The kernel of the integral equation for the LDLIV process

The stable form of the kernel in Eq. 3 derived by Buonocore et al., (1987), although it characterizes many of the processes of interest to decision modelers, is not applicable to either the LDLIV process or the Wiener process with reflecting boundary at *r*. This is because both processes have a boundary — a so-called *regular* boundary — at which the equation become singular. A regular boundary is a point that can be attained, but not exceeded, by a process starting in the interior of the state space, and conversely, a process starting on the boundary can attain a point on the interior of the space at a later time (Karlin and Taylor 1981, p. 234). For the Wiener process, *r*, *r* < *z*, is a regular boundary; for the LDLIV process, 0 is a regular boundary.^3^ Although the general form of the kernel function derived by Buonocore et al. does not apply to either of these processes, Giorno et al., (1989a) showed that it is nevertheless possible to derive kernel functions for Eq. 3 in which the resulting discrete approximations are stable. I draw heavily on their results throughout the remainder of this article.

For the LDLIV process, Giorno, Nobile, Ricciardi, and Sato (1989a, Eq. 6.4) showed the kernel is

4 $$ \begin{array}{@{}rcl@{}} {\Psi}[a(t), t | z, \tau] & = & \frac{p}{\sigma[e^{p(t-\tau)} - 1]} \exp\left\{-\frac{p[a(t) + ze^{p(t-\tau)}]}{\sigma[e^{p(t-\tau)} - 1]}\right\} \\&& \times\left[\frac{a(t)e^{-p(t - \tau)}}{z}\right]^{(q - \sigma)/2\sigma} \\ && \times \left\{\left[a^{\prime}(t) - \frac{pa(t)e^{p(t - \tau)}}{e^{p(t - \tau)} - 1} + k(t)\right]\right. \\ && \times \left.I_{q/\sigma -1}\left[\frac{2p\sqrt{a(t)ze^{p(t - \tau)}}} {\sigma[e^{p(t - \tau)} - 1]}\right]\right. \\ && + \left.\frac{p\sqrt{a(t)ze^{p(t - \tau)}}}{e^{p(t - \tau)} - 1} I_{q/\sigma}\left[\frac{2p\sqrt{a(t)ze^{p(t-\tau)}}}{\sigma[e^{p(t-\tau)} -1]}\right]\right\}. \end{array} $$

In Eq. 4, *I*_*ν*_(⋅), is a modified Bessel function of the first kind of order *ν* (Abramowitz and Stegun 1965, p. 374), and *k*(*t*) is a function that is chosen to make the kernel of the integral equation nonsingular (Giorno et al., 1989a, Eq. 6.7), which is of the form

5 $$ k(t) = \frac{1}{2}\left[pa(t) + q - \frac{\sigma}{2} - a^{\prime}(t)\right], $$

where $a^{\prime }(t)$ is the derivative of the absorbing boundary *a*(*t*). Here I am concerned exclusively with the constant boundary case, *a*(*t*) ≡ *a*, in which case the terms $a^{\prime }(t)$ in Eqs. 4 and 6 are both zero.^4^

I assume that the decision process takes the form of a race between LDLIV processes, each of which accumulates evidence for a given response alternative. The joint decision time-accuracy distributions are then given by the race model equation, in which the joint density is the product of the first-passage time density for the first-finishing process multiplied by the survivor function for the other process (or processes in *n*-alternative cases). Denoting the first passage time density for process *i* by *g*_*i*_(*t*), the distribution function by *G*_*i*_(*t*), and the survivor function by $\bar {G}_{i}(t)$, where $\bar {G}_{i}(t) = 1 - G_{i}(t)$, the joint densities are

6 $$ g_{\text{race}}(t, i) = g_{i}(t) \bar{G}_{j;  j\ne i}(t);~~i,j = 1, 2. $$

Models of the form of Eq. 6 have a long history in the RT literature. An advantage of this representation is that it carries over easily to *n*-choice decisions, but I restrict myself here to the two-choice case. I discuss how I parameterize the model to fit it to data in a subsequent section.

## The kernel of the integral equation for the reflecting wiener process

A race model based on LDLIV processes satisfies all of the three preceding principles for a neurally-plausible decision model. A model based on racing reflecting Wiener processes satisfies Principles 1 and 3 but relaxes Principle 2 (saturation at high intensities). Giorno et al. (1989a, Eq. 5.14) showed that it was possible to derive a stable kernel in Eq. 3 for a Wiener process with drift rate, *μ*, diffusion rate, *σ*^2^, and a reflecting boundary at *r*, *r* ≤ *z*, using arguments like those used to derive Eq. 4. For the reflecting Wiener process the kernel takes the form

7 $$ \begin{array}{@{}rcl@{}} {\Psi}[a(t), t | z, \tau] & = & \frac{1}{2\sigma\sqrt{2\pi(t - \tau)}}\left[a^{\prime}(t) - \frac{a(t) - z}{t - \tau}\right] \\& \times&\exp\left\{-\frac{[a(t) - z - \mu(t-\tau)]^{2}}{2\sigma^{2}(t - \tau) }\right\} \\ & + & \frac{1}{2\sigma\sqrt{2\pi(t - \tau)}} \\& \times&\left[a^{\prime}(t) - \frac{a(t) + z - 2r - 2\mu(t - \tau)}{t - \tau}\right] \\ & \times & \exp\left\{-\frac{2\mu(z - r)}{\sigma^{2}}\right\}\\&\times&\exp\left\{-\frac{[a(t) + z - 2r - \mu(t - \tau)]^{2}}{2\sigma^{2}(t - \tau)} \right\} \\ & - & \frac{\mu[a^{\prime}(t) + \mu]}{\sigma^{2}} \exp\left\{\frac{2\mu[a(t) - r]}{\sigma^{2}}\right\}\\&\times&{\Phi}\left[-\frac{a(t) + z - 2r + \mu(t - \tau)}{\sigma\sqrt{t - \tau}}\right], \end{array} $$

where Φ(⋅) is the normal distribution function.^5^ As in Eq. 4, in the constant boundary case that we are concerned with here, *a*(*t*) ≡ *a* and the terms involving $a^{\prime }(t)$ are zero.

**Fig. 2:**
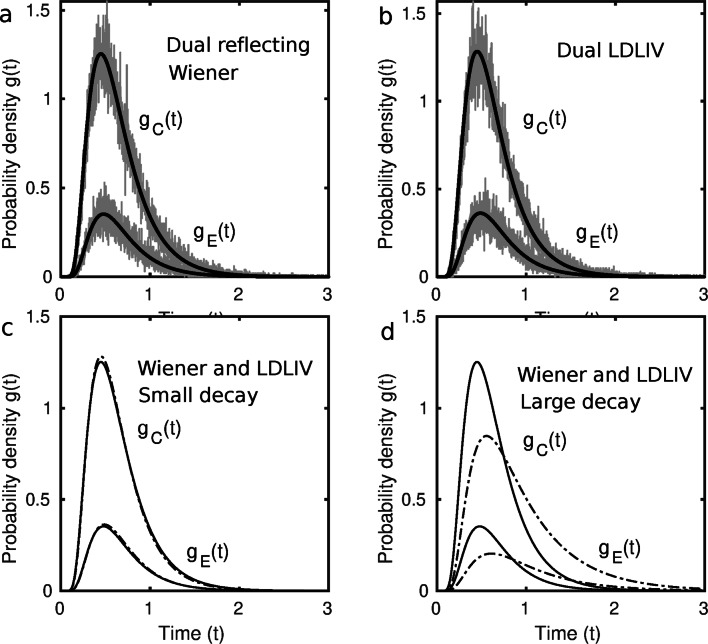
Simulated and predicted joint density functions for correct responses, *g*_*C*_(*t*), and errors, *g*_*E*_(*t*), for dual reflecting Wiener and LDLIV models. Each of the simulations was based on 100,000 trials using a time step of 0.001 s. (a) Dual reflecting Wiener model with parameters *μ*_1_ = 1.75, *μ*_2_ = 0.5, *a*_1_ = 1.5, *a*_2_ = 1.5, *σ* = 1.0, *z* = 0, *r* = − 0.1. (b) Dual LDLIV model with parameters *q*_1_ = 1.5, *q*_2_ = 0.75, *a*_1_ = 1.2, *a*_2_ = 1.2, *σ* = 1.0, *p* = − 0.01, *z* = 0.1. (c) Comparison of the reflecting Wiener and LDLIV models with parameters as in (a) and (b). (d) Comparison of the reflecting Wiener and LDLIV models with parameters as in (a) and (b) except with large LDLIV decay (*p* = − 1.0). In (c) and (d) the continuous curves are the reflecting Wiener model and the dashed curves are the LDLIV model

Figure 2 shows predictions for models with racing LDLIV and reflecting Wiener processes. The top panels compare predictions for the models obtained using the integral equation method with simulations of them using the Euler method (Brown, Ratcliff, & Smith, 2006). The latter approximates the diffusion process with a discrete time, Gaussian-increments, random walk. Each of the simulations was based on 100,000 trials with a step size of 0.001 s. The predictions were computed using Eq. A3 using a time step of 0.01 s, which sufficed to provide good agreement with the simulations. The simulations show that the integral-equation method is an effective way to evaluate models of this kind. Smith and Ratcliff (2022) give further examples of simulated and integral-equation predictions for time-varying Wiener and OU processes.

The bottom panels of Fig. 2 compare the dual reflecting Wiener and LDLIV models to each other. Figure 2c compares the predictions when the LDLIV decay parameter is small (*p* = − 0.01) and Fig. 2d compares them when it is large (*p* = − 1.0). The parameters of the models in Fig. 2a and b were chosen by eye to try to make the predictions as close to each other as possible. As Fig. 2c shows, when the parameters were chosen in this way and decay was small, the models are in close agreement. When decay is increased, the mean and the variance of the distributions increase and the model predicts heavy right tails. The behavior of the model is therefore similar to the OU model, which predicts similar changes in the distribution shape as decay is increased.

## The two-barrier and single-barrier Wiener processes

For consistency of implementation I used the integral-equation method to obtain predictions for the two-barrier Wiener model. Ratcliff and Smith (2004) compared the infinite-series method and the integral-equation method and found their predictions were in close agreement. Smith and Ratcliff (2022) presented integral-equation solutions for the Wiener model with time-varying drift and diffusion rates and decision boundaries. I used a version of their code with drift and diffusion rates held constant, augmented with variability in drift rates and starting points, to carry out the model fits described below.

The first-passage time density for the single-boundary Wiener process has a simple closed-form solution that is well known in the literature (Karlin & Taylor 1975, p. 363) where it is variously referred to as the Wald or the inverse-Gaussian distribution. The first-passage time density has the form

8 $$ g(a, t | z, 0) = \frac{a - z}{\sigma\sqrt{2\pi t^{3}}} \exp\left[-\frac{(a - z - \mu t)^{2}}{2\sigma^{2} t}\right]. $$

The meaning of the parameters in this model is the same as in the reflecting Wiener model of Eq. 7. Equation 8 has a long history in the psychological literature in modeling both simple and choice RT (e.g., Emerson, 1970; Heathcote, 2004; Matzke & Wagenmakers, 2009; Schwarz, 2001; 2002). It is possible to derive a closed-form expression for the marginal first-passage density for this process when the starting point, *z*, is uniformly distributed across trials rather than constant (Logan et al., 2014; Tillman et al., 2020) but, again, for consistency of implementation, I evaluated the effects of starting point variability numerically, as discussed below.

## Method

### Experimental studies

I compared the dual LDLIV and reflecting Wiener models, the racing Walds model, and the standard diffusion model using the data from the numerosity discrimination study of Ratcliff (2008) and the attentional cuing study of Smith et al., (2004). Smith and Ratcliff (2022) used the data from these studies to compare urgency-gating and collapsing boundaries models to the standard diffusion model. The version of the standard diffusion model I used to fit the Ratcliff (2008) data was slightly more general than the one considered by them in that it allowed nondecision times to vary with experimental instructions, for reasons described below.

The participants in Ratcliff’s (2008) study were asked to decide whether the number of randomly-placed dots in a 10 × 10 grid was greater or less than 50. There were eight nominal discriminability conditions, in which the numbers of dots were: 31-35, 36-40, 41-45, 46-50, 51-55, 56-60, 61-65, and 66-70, crossed with speed versus accuracy instructions. On half the trials participants were instructed to respond rapidly and on the other half they were told to respond accurately and they were given feedback to encourage them to perform as instructed. Data were collected from 19 college-aged participants and 19 older participants who performed both a standard RT task, in which they responded as soon as they had sufficient evidence, and a response-signal task, in which they responded to a random external deadline. Following Smith and Ratcliff (2022), I restrict my analysis to the younger participants and the standard RT task. After eliminating fast and slow outliers, there were about 850 valid trials in each cell of the design, yielding around 13,600 valid trials per participant.

Participants in the Smith et al., (2004) study performed an attentional cuing task in which low-contrast Gabor patches were presented for 60 ms at either a cued location, which was indicated by a 60 ms, flashed peripheral cue 140 ms before the stimulus, or at one of two uncued locations. On each trial, participants decided whether the orientation of the patch was vertical or horizontal. In one condition of the experiment the stimuli were backwardly masked with high-contrast checkerboards and in the other condition they were briefly flashed and then extinguished. Data were collected from six highly-practiced participants who performed the task at five different levels of contrast, which were chosen for each participant individually during practice to span a range of performance from just above chance (≈ 55*%* correct) to near-perfect (≈ 95*%* correct). Participants were encouraged to be as accurate as possible but not to deliberate for too long and were given auditory accuracy feedback on each trial. There were 400 valid trials for each participant in each cell of the Cue × Mask × Discriminability design, yielding 8000 trials per participant. The reader is referred to Smith and Ratcliff (2022) and the original articles for more details on the two studies.

## Results

In the standard diffusion model, evidence accumulation is modeled by a Wiener diffusion process between absorbing boundaries that represent decision criteria for the two responses. In addition to within-trial, diffusive variability there are three sources of across-trial variability: variability in drift rates, starting points, and nondecision times (Ratcliff & McKoon, 2008). Drift rate is normally distributed with mean *ν* and standard deviation *η*; starting point is uniformly distributed with range *s*_*z*_, and nondecision time, *T*_er_, is uniformly distributed with range *s*_*t*_. Variability in drift rate and starting point allows the model to predict the ordering of correct responses and errors; variability in nondecision time allows it to better predict the leading edges of RT distributions under speed-stress conditions when accuracy is high and RTs are short. Typically, error RTs tend to be longer than RTs for correct responses when the task is difficult and accuracy is stressed and shorter when the task is easy and speed is stressed (Luce, 1986, p. 233).

In the dual-diffusion models, each accumulator has its own drift rate, which may be unrelated or be constrained in some way. These models raise questions about how to parameterize drift rates in a way that is theoretically principled, parsimonious, and flexible enough to account for the relationships found in empirical data. A common assumption is to assume that the drift rates or the mean drift rates sum to a constant across stimulus difficulty levels (Ratcliff & Smith, 2004; Ratcliff et al., 2007; Usher & McClelland, 2001). Although it is possible to implement a dual-diffusion model in which the drift rates are normally distributed — Smith and Ratcliff (2009) proposed a dual-diffusion model with reflecting OU processes and normally-distributed, negatively-correlated drift rates — a more natural assumption is to constrain drift rates to be positive. In the LDLIV process the positivity constraint is needed to avoid the *q* ≤ 0 exit boundary properties described in Footnote 3.

I assumed the drift rates in the dual-diffusion models were lognormally distributed (i.e., random variables whose logarithms are normally distributed), with dispersion parameters *η*_1_ and *η*_2_ for the accumulators associated with correct and error responses, respectively. The drift rates in stimulus condition *j* were distributed as

9 $$ \begin{array}{@{}rcl@{}} \mu_{j1} & \sim & \nu_{j1} \exp(\eta_{1}\xi_{1})\! \! = \pi_{j}\nu_{\text{sum}} \exp(\eta_{1}\xi_{1}) \\ \mu_{j2} & \sim & \nu_{j2} \exp(\eta_{2}\xi_{2})\!\! =\! \! (1 - \pi_{j})\nu_{\text{sum}} \exp(\eta_{1}\xi_{1}); j\!\!  =\! \! 1\! ,\! 2,\! \ldots, \end{array} $$

where *ξ*_1_ and *ξ*_2_ are independent standard normal variates and *π*_*j*_, 0 ≤ *π*_*j*_ ≤ 1, is a mixing parameter that constrains *ν*_sum_ to be constant across stimulus conditions. With this parameterization the scaling factors *ν*_*j*1_ and *ν*_*j*2_ are the medians of the lognormal distributions, so Eq. 9 constrains the sums of the median drift rates in the two accumulators to be constant. A constraint on the medians could be seen as a natural one when the drift rate distributions are positively skewed, but I adopted it for computational convenience rather than because of a theoretical preference for medians over means. Like a constraint on the means, the constraint on the medians almost halves the number drift rate parameters that are needed to fit the model to data. Parameterized in this way, the LDLIV model requires five drift rate parameters to fit the four discriminability conditions of the Ratcliff et al. (2008) data and six to fit the five discriminability conditions of the Smith et al., (2004) data. In the notation of the LDLIV model, *μ* ≡ *q*, the stimulus-dependent part of drift rate.

In the case of the model with racing Walds (single-boundary Wiener processes with no reflecting boundaries), Tillman et al., (2020) reported the model can successfully account for slow errors without across-trial variability in drift rates because of its race-model structure, which naturally predicts slow errors. Indeed, if combined with the usual assumption of normally-distributed drift rates then the model would predict defective RT distributions because on some trials the drift rate would be away from the boundary and the probability the process would ever terminate would be less than 1.0. To evaluate the generality of Tillman et al.’s findings, I implemented the racing Walds model, as they did, without drift-rate variability. Initially, I imposed a constraint that the drift rates should sum to a constant, but found it performed poorly relative to the other dual diffusion models. I therefore allowed the drift rates for the two accumulators to vary freely and independently across conditions, which gave the model a similar number of free parameters to the other models. The fits I report are for the model with unconstrained drift rates.

There has been debate in the literature about whether speed-accuracy manipulations affect only decision criteria, as they are theoretically assumed to do, or whether they affect other processes as well. Several researchers have reported that speed-accuracy instructions affect nondecision times in addition to decision criteria (Arnold, Bröder, & Bayden, 2015; de Hollander et al., 2016; Donkin, Brown, Heathcote, & Wagenmakers, 2011; Dutilh et al., 2019; Huang et al., 2015)and others have reported they also affect mean drift rates or drift-rate variability (Donkin et al., 2011; Heathcote & Love, 2012; Ho et al., 2012; Rae, Heathcote, Donkin, Averell, & Brown, 2014; Starns, Ratcliff, & White, 2012). Smith and Lilburn (2020) suggested that these so-called violations of selective influence may reflect time-inhomogeneity in the drift and diffusion rates, and reanalyzed the random dot motion data of Dutilh et al. using a time-inhomogeneous diffusion model that captured the data well. Here I restrict myself to time-homogeneous versions of the models for the sake of computational tractability. Smith and Ratcliff (2022) reported a reanalysis of the Ratcliff (2008) numerosity data using models with a single *T*_er_ parameter but I have instead allowed *T*_er_ to vary with instructions as this produces substantial improvements in fits for the standard diffusion model. Whether these so-called violations of selective influence are best characterized as changes in the time of onset of evidence accumulation or as time-varying drift and diffusion rates is a question that is outside the scope of this article. My aim here is the narrower one of comparing the standard diffusion model and the racing diffusion models under conditions that yield the best accounts of the data for each model. The parameters of the models and their interpretations are summarized in Table 1.

**Table 1:** Parameters of the decision models

Parameter	Symbol
Single diffusion boundary separation speed	*a*_*s*_
Single diffusion boundary separation accuracy	*a*_*a*_
Dual diffusion boundary accumulator *i*, speed	*a*_*is*_
Dual diffusion boundary accumulator *i*, accuracy	*a*_*ia*_
Single and dual diffusion starting point speed	*z*_*s*_
Single and dual diffusion starting point accuracy	*z*_*a*_
Mean drift rate, accumulator *i* condition *j*	*ν*_*ji*_
Sum of median drift rates	*ν*_sum_
Drift rate proportionality condition *i*	*π*_*i*_
Nondecision time, speed	*T*_er, *s*_
Nondecision time, accuracy	*T*_er, *a*_
Drift rate variability	*η*
Starting point variability	*s*_*z*_
Nondecision time variability	*s*_*t*_
Infinitesimal standard deviation	*σ*
LDLIV decay rate	*p*
Wiener diffusion reflecting boundary	*r*

### Fitting method

How best to evaluate RT models empirically remains a subject of active debate, with classical and Bayesian, hierarchical and nonhierarchical methods all being widely used and advocated. The variety of methods used in the recent blinded validity study of Dutilh et al., (2019) highlights the diversity of practice among researchers in the area. Ratcliff and Childers (2015) carried out a parameter recovery study using the diffusion model in which they compared classical and hierarchical Bayesian methods and found that hierarchical Bayesian methods improved parameter recovery for small samples (i.e., small numbers of trials in each experimental condition for each participant), but when samples were large, there was little difference between them and classical methods.

I chose to use methods similar to those used in the original studies of Ratcliff (2008) and Smith et al., (2004) and in the reanalysis of their data by Smith and Ratcliff (2022). I minimized the likelihood-ratio chi-square statistic (*G*^2^) for the response proportions in the bins formed by the .1, .3, .5, .7, and .9 RT quantiles for the distributions of correct responses and errors. When bins are formed in this way there are a total of 12 bins (11 degrees of freedom) in each pair of joint distributions of correct responses and errors. The resulting *G*^2^ may then be written as a function of the observed and expected proportions in the bins as

10 $$ G^{2} = 2 \sum\limits_{i=1}^{M} n_{i} \sum\limits_{j=1}^{12} p_{ij} \log\left( \frac{p_{ij}}{\pi_{ij}}\right). $$

In Eq. 10, *p*_*ij*_ and *π*_*ij*_ are, respectively, the observed and predicted response proportions in the bins bounded by the RT quantiles and “log” is the natural logarithm. The inner summation extends over the 12 bins formed by each pair of joint distributions and the outer summation extends over the *M* experimental conditions. For the numerosity study, *M* = 16 (2 Instruction conditions × 8 Dot proportions). For the cuing study, *M* = 5 (5 contrast conditions for each cell of the Cue × Mask experimental design). The quantity *n*_*i*_ is the number of experimental trials on which each joint distribution pair was based. For the numerosity study *n*_*i*_ ≈ 850 and for the cuing study *n*_*i*_ = 400. I fit the models to the individual participants’ data by minimizing *G*^2^ using the Nelder-Mead simplex algorithm (Nelder & Mead, 1965) as implemented in Matlab (fminsearch). The fit statistics I report are the minimum *G*^2^ values obtained from six runs of simplex using randomly perturbed estimates from the preceding run as the starting point for the next run. One of my reasons for preferring fits at the individual level using classical methods to hierarchical Bayesian methods is the latter — as least as they have been implemented in the literature to date — tend to obscure the quality of the fits and the variation in comparative model performance at the individual participant level.

To compare models with different numbers of parameters, I used standard model selection methods based on the Akaike information criterion (AIC, Akaike, 1974) and the Bayesian information criterion (BIC, Schwarz, 1978). The first of these statistics is derived from classical principles whereas the second is Bayesian, but I use them in the spirit in which they are typically used in the modeling literature, as penalized likelihood statistics that impose more or less severe penalties on the number of free parameters in a model. As is well known, the AIC tends to gravitate towards more complex models with increasing sample sizes more quickly than does the BIC (Kass & Raftery, 1995). For binned data, the AIC and BIC may be written as

11 $$ \begin{array}{@{}rcl@{}} AIC & = & G^{2} + 2k \end{array} $$

12 $$ \begin{array}{@{}rcl@{}} BIC & = & G^{2} + k\log N, \end{array} $$

where *k* is the number of free parameters in the model and $N = {\sum }_{i} n_{i}$ is the total number of observations on which the fit statistic was based.

### Numerosity study (Ratcliff, 2008)

In the following tables, I use the identifiers “DIFF” to denote the standard diffusion model, “LDLIV” to denote the linear-drift, linear-infinitesimal variance model, “WNR” to denote the reflecting Wiener model, and “WLD” to denote the racing Walds model. Table 2 shows mean *G*^2^ statistics for each model, averaged over the 19 participants, together with the corresponding mean AIC and BIC values.

**Table 2:** Model fits for the numerosity study

Model	*G*^2^	*k*	*df*	AIC	BIC
DIFF	656.1	13	163	682.1	779.8
LDLIV	589.5	17	159	623.5	751.3
WNR	805.9	16	160	837.9	958.1
WLD	826.8	16	160	864.8	979.1

On average, the LDLIV model was the best-performing of the models by a substantial margin. The next best model was the diffusion model, followed by the reflecting Wiener model, followed by the racing Walds model. This pattern is mirrored at the individual participant level, as shown in Table 3, which shows the number of participants for whom each of the models was better than each of its competitors, as assessed by either the AIC or the BIC. The diffusion model was better than the LDLIV model for just under half the participants (9/19), but was better than either of the other dual diffusion models for the majority of participants by either criterion. The LDLIV model was better than either of the other two dual diffusion models for a substantial majority of participants. Of those two models, the reflecting Wiener process model was better than the racing Walds model for most participants.

**Table 3:** Comparative fits for the numerosity study by AIC and BIC

	#AIC				#BIC			
Model	DIFF	LDLIV	WNR	WLD	DIFF	LDLIV	WNR	WLD
DIFF	−	9	14	15	−	9	14	15
LDLIV		−	16	17		−	16	17
WNR			−	12			−	12

Number of participants for whom the row model was better than the column model (out of 19)

In comparison, the *G*^2^ statistics for models with a single *T*_er_ parameter were 820.5, 621.7, 857.9, and 888.8, for the DIFF, LDLIV, WNR, and WLD models, respectively. The average improvement in fit when *T*_er_ varied with instructions ranges from 5% to 20%, with the largest improvement for the standard diffusion model. Table 4 shows the estimated parameters for the four models. Consistent with the observation that the standard diffusion model showed a large improvement in fit with variable *T*_er_, it also showed the largest difference in the estimates of *T*_er, *s*_ and *T*_er, *a*_ (270 ms vs. 282 ms, respectively). The corresponding estimates for the the LDLIV model were 264 ms and 267 ms. The estimates of *T*_er, *a*_ for the WNR and WLD models were shorter than those of *T*_er, *s*_ rather than longer, suggesting the extra parameter in these models was simply describing random variation rather than essential structure in the data.

**Table 4:** Model parameters for the numerosity study

	*a*_*s*_	*a*_*a*_	*ν*_1_	*ν*_2_	*ν*_3_	*ν*_4_			
DIFF	0.078	0.126	0.510	0.417	0.281	0.094			
	*z*_*s*_	*z*_*a*_	*η*	*s*_*z*_	*T*_er, *s*_	*T*_er, *a*_	*s*_*t*_		
	0.040	0.065	0.130	0.045	0.270	0.282	0.133		
LDLIV	*a*_*s*1_	*a*_*s*2_	*a*_*a*1_	*a*_*a*2_	*π*_1_	*π*_2_	*π*_3_	*π*_4_	
	0.503	0.522	0.759	0.762	0.975	0.879	0.740	0.576	
	*ν*_sum_	*z*	*p*	*η*_1_	*η*_2_	*s*_*z*_	*T*_er, *s*_	*T*_er, *a*_	*s*_*t*_
	4.412	0.208	2.428	0.216	0.096	0.262	0.264	0.267	0.113
WNR	*a*_*s*1_	*a*_*s*2_	*a*_*a*1_	*a*_*a*2_	*π*_1_	*π*_2_	*π*_3_	*π*_4_	
	0.650	0.682	1.255	1.268	0.994	0.924	0.767	0.583	
	*ν*_sum_	*r*	*η*_1_	*η*_2_	*s*_*z*_	*T*_er, *s*_	*T*_er, *a*_	*s*_*t*_	
	6.195	− 1.014	0.288	0.212	0.512	0.253	0.237	0.101	
WLD	*a*_*s*1_	*a*_*s*2_	*a*_*a*1_	*a*_*a*2_	*ν*_1_	*ν*_2_	*ν*_3_	*ν*_4_	
	0.570	0.603	1.056	1.080	0.014	0.137	0.761	1.749	
	*ν*_5_	*ν*_6_	*ν*_7_	*ν*_8_	*s*_*z*_	*T*_er, *s*_	*T*_er, *a*_	*s*_*t*_	
	5.379	4.738	3.854	2.724	0.389	0.248	0.228	0.102	

Diffusion model parameters scaled with *σ* = 0.1; other models scaled with *σ* = 1.0

**Fig. 3:**
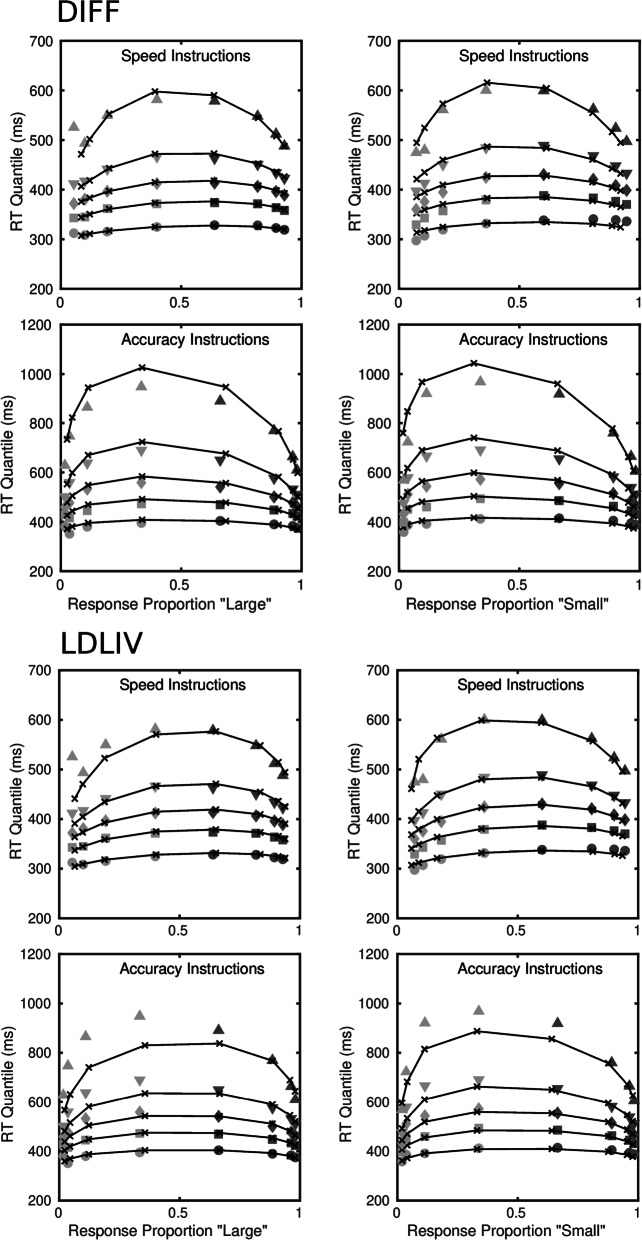
Quantile probability functions for “large” and “small” responses for speed and accuracy conditions for the diffusion model, DIFF, and the LDLIV model fitted to the Ratcliff (2008) numerosity data. The quantile RTs in order from the bottom to top are the .1, .3, .5, .7, and .9 quantiles (circles, squares, diamonds, inverted triangles, upright triangles, respectively). The dark gray symbols are the quantiles for correct responses and the light gray symbols are the quantiles for errors. The continuous curves and *x*’s are the predictions from the model. For the data and models the quantile RTs are plotted on the *y*-axis against the observed and predicted response proportions on the *x*-axis

**Fig. 4:**
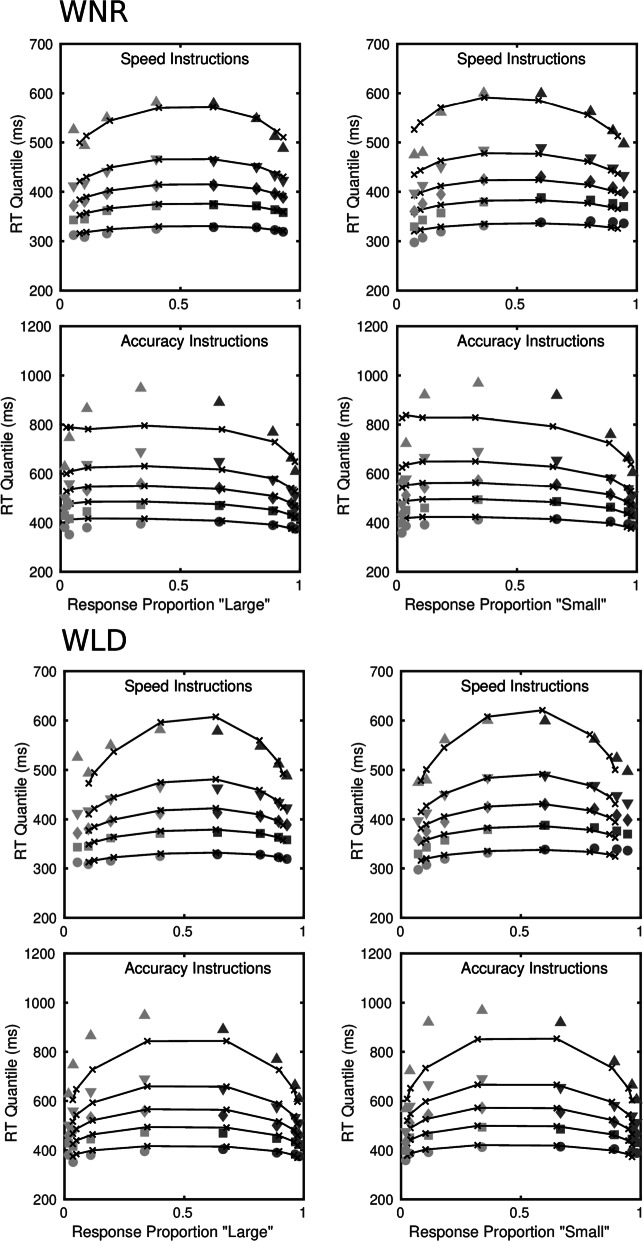
Quantile probability functions for “large” and “small” responses for speed and accuracy conditions for the reflecting Wiener model, WNR, and the racing Walds model, WLD, fitted to the Ratcliff (2008) numerosity data. The quantile RTs in order from the bottom to top are the .1, .3, .5, .7, and .9 quantiles (circles, squares, diamonds, inverted triangles, upright triangles, respectively). The dark gray symbols are the quantiles for correct responses and the light gray symbols are the quantiles for errors. The continuous curves and *x*’s are the predictions from the model. For the data and models the quantile RTs are plotted on the *y*-axis against the observed and predicted response proportions on the *x*-axis

Of the four models, the diffusion model is the most parsimonious, in the sense of requiring the fewest parameters to account for the data. Even with constraints on the drift rates, the LDLIV and the reflecting Wiener process models required three or four more parameters because of the need to parameterize the mean drift rate and the drift rate variability for the two accumulators separately. The racing Walds model is able to characterize the data without drift rate variability, as Tillman et al., (2020) pointed out, but it required a total of eight free drift rate parameters in order to do so and was clearly the worst-performing of the models. Figure 3 shows fits of the diffusion and LDLIV models to the group data; Fig. 4 shows the corresponding fits for the reflecting Wiener and racing Walds models. The fits are shown as quantile-probability plots, in which quantiles of the RT distributions for correct responses and errors are plotted against the choice probabilities for a range of stimulus difficulties. Readers who are unfamiliar with this way of representing model fits are referred to Ratcliff and Smith (2004) or Ratcliff and McKoon (2008), among other other sources, for an account of how they are constructed. The data in Figs. 3 and 4 are quantile-averaged group data and the fitted values are quantile-averaged individual fits. The fits for the diffusion model in the upper part of Fig. 3 are the same as those in Fig. 3 in Smith and Ratcliff (2022).

The most obvious difference between the two models is in the tails of the predicted RT distributions. One of the effects of combining racing processes in a dual diffusion architecture is that the predicted RT distributions for the fastest-finishing process will be less skewed and more symmetrical than the distributions of either of the component processes in isolation. This is because slow responses occur only if both processes are slow to finish. By the race model equation (Eq. 6), an RT longer than a given *t* is equal to the product of the probabilities that both processes take longer than *t* to finish (i.e., the product of their survivor functions), which will be less than the finishing time probabilities of either process individually. The reduction in skewness is offset by the presence of decay, denoted *p* for the LDLIV model in Eq. 2, and estimated to be, on average, *p* = 2.428 in Table 4. This interaction between the architecture of the model and the effects of decay is likely why Ratcliff and Smith (2004) found decay to be zero in a single-process OU model but Smith and Ratcliff (2009) found decay was nonzero in a dual-diffusion OU model. The difference between single-process and dual-process models is evident in the distribution tails for the diffusion and LDLIV models in Fig. 3, as represented by the .9 quantile function (the topmost line in the plot). The diffusion model accurately predicts the .9 quantile in the speed condition and somewhat overpredicts it in the accuracy condition. The LDLIV model slightly underpredicts the .9 quantile function in the speed condition and underpredicts it more severely in the accuracy condition, especially for the error distributions. The tendency for the diffusion model to overpredict the .9 quantile function in the accuracy condition was reduced by allowing *T*_er_ to vary with instructions. Indeed, the most obvious qualitative difference between models with one and two *T*_er_ parameters was in how well the .9 quantile functions in the accuracy condition were predicted.

The tendency for dual diffusion models to underpredict the tails of RT distributions is particularly pronounced in the reflecting Wiener model, shown in the upper panels of Fig. 4. The reflecting Wiener process model was worse than the LDLIV model for a substantial majority of the participants (16/19 by either criterion). The reflecting Wiener model captured the shapes of the RT distributions fairly well in the speed condition but missed substantially in the accuracy condition. The pattern of misses is similar to that for the LDLIV model, but is magnified because the Wiener model does not have a decay term to offset the tendency for dual diffusion models to predict RT distributions that are more symmetrical than those predicted by the standard model.

Although the reflecting Wiener model was worse than the LDLIV model, it was nevertheless better than the racing Walds model for the majority of participants (12/19 by either criterion). Although the latter model is in principle able to predict slow errors by virtue of its race model structure, it failed to do so in the accuracy condition of this experiment. Rather, the predicted quantile-probability functions in the accuracy condition in Fig. 4 are almost symmetrical across the vertical midline, indicating the model is predicting essentially the same RT distributions for correct responses and errors. There are two reasons why the model might have failed to capture the slow error pattern in these data. The first is that it may be unable to capture the slow error pattern if the range of stimulus discriminabilities and associated accuracy varies widely. The second is that it may be unable to capture the slow error pattern when discriminability is crossed with speed versus accuracy instructions – even when *T*_er_ is allowed to vary with instructions, as was the case here.

Tillman et al., (2020) reported a simulation showing that the magnitude of the slow error effect predicted by the model increases with decision criterion and, consequently, increases monotonically with accuracy (their Fig. 3). However, they did not characterize the way in which the magnitude of the effect varies with drift rate and it is not obvious from their results that the model can capture a slow error pattern of the complexity of the one shown in Figs. 3 and 4. They reported a fit of the racing Walds model to the data of Ratcliff and Rouder (1998), which crossed stimulus discriminability with speed versus accuracy instructions, but they fit the mean RTs only. They also reported a fit to the RT distribution data of Rae et al., (2014), which varied speed versus accuracy instructions, but used only a single stimulus discriminability level, and even in this relatively unconstrained case the model appears to be failing to capture the shapes of the error distributions (their Fig. 8). As a further, more constrained, test they fit the model to a data from a lexical decision experiment reported by Ratcliff and Smith (2004) that collected RT and accuracy data for high-frequency, low-frequency, and very-low-frequency words and pseudowords, each of which was modeled with its own drift rate. It is difficult to infer the quality of the fit from Tillman et al.’s figure (their Fig. 6), which show joint cumulative distributions rather than quantile probability functions (see Luce (1986, pp. 17–20), for a relevant discussion on this point), but it suggests a significant failure to fit in some conditions, most obviously for error responses to pseudowords. Overall, the data of Ratcliff (2008) represent an appreciably greater challenge for the model than do the data sets that Tillman et al. considered and, as I have shown, on the Ratcliff data the model does not fare well. Our next application of the model to the attention cuing study of Smith et al., (2004) speaks further to the reasons for the model’s failure.

As well as differing in whether there was drift-rate variability, the reflecting Wiener and racing Walds models also differed in whether or not the process was constrained by a lower reflecting boundary. Unlike the reflecting Wiener model, the racing Walds model does not have a reflecting boundary to limit the excursions of the process away from the decision boundary. A single-boundary Wiener process, as characterized by the Wald distribution, can make arbitrarily large excursions away from the boundary with finite probability, which often leads to long finishing times. Indeed, if the drift rates are negative or zero then the probability that the process will never finish is greater than zero and the RT distributions will be defective, as noted previously.

To evaluate whether drift-rate variability or the reflecting boundary was the primary determinant of the difference between the two models, I refit the reflecting Wiener model with the reflecting boundary set to $r = -1.5\max \limits (a_{1a}, a_{2a})$, that is, as a multiple of the largest of the two accuracy criteria. I conjectured, based on the estimates in Table 4, which show *r* set quite far from the starting point, that the reflecting boundary was contributing relatively little to the quality of the fit. The multiple of 1.5 was arbitrary, but served to minimize the effects of the boundary while avoiding numerical instabilities in the computation of the kernel. With *r* constrained in this way, the performance of the model was essentially unaltered. The mean *G*^2^ was 805.5, which is virtually identical to the *G*^2^ with *r* freely-varying in Table 2. (The very slight improvement in fit found with *r* constrained is due to the constrained model’s better convergence properties.) The comparative performance of the constrained model at the individual level was identical to that of the model with *r* freely-estimated in Table 3.

The large negative estimates of *r* in Table 4 contrast with Smith and Ratcliff’s (2009) dual-diffusion model, which consists of racing OU processes with negatively correlated, normally-distributed, drift rates and reflecting lower boundaries. They estimated the reflecting boundaries to be just below the starting points (again zero). Because of the negatively correlated drift rates in their model, error processes tend to drift towards the lower reflecting boundary, which prevents large negative excursions in the accumulating evidence, allowing the model to correctly predict distributions of error RTs. In the reflecting Wiener model, the drift rates are lognormally distributed and constrained to be positive, so all of the processes drift upwards and large negative excursions in the accumulating evidence are comparatively infrequent. Consequently, the reflecting boundary in the Wiener model has relatively little effect on model performance.

From the quantile-probability plot, the reasons for the better performance of the LDLIV model compared to the diffusion model are not particularly obvious, but they appear to lie in the relative ability of the models to account for faster responses. One of the unique features of the LDLIV model not shared with the other models is in the effects of starting point and starting point variability. The Wiener process models — including the diffusion model — are spatially homogeneous. Changing the starting point simply translates the process on the real line, $\mathbb {R}$, and is tantamount to relabeling the decision boundaries relative to the new starting point. In contrast, the LDLIV model with a nonzero starting point is qualitatively different from one with a zero starting point because the diffusion rate in Eq. 2 depends on the position of the process in the evidence space, $\mathbb {R^{+}}$. A process with a starting point of *z* > 0 will begin to diffuse towards the boundary more rapidly than one with a starting point of *z* ≈ 0. Table 4 shows that the estimated starting point in the LDLIV model was between a half and a third of the way towards the decision boundary in the speed and accuracy conditions, respectively. Unlike the other models, the starting point in the LDLIV model affects not only the distance the process has to travel in order to reach a boundary but also the speed with which it does so. In the other models, the distance to the boundary and the speed with which the process travels are independent of each other. This dependency seems to be what gives the model an advantage under conditions in which speed is stressed.

### Spatial cuing study (Smith et al., 2004)

**Fig. 5:**
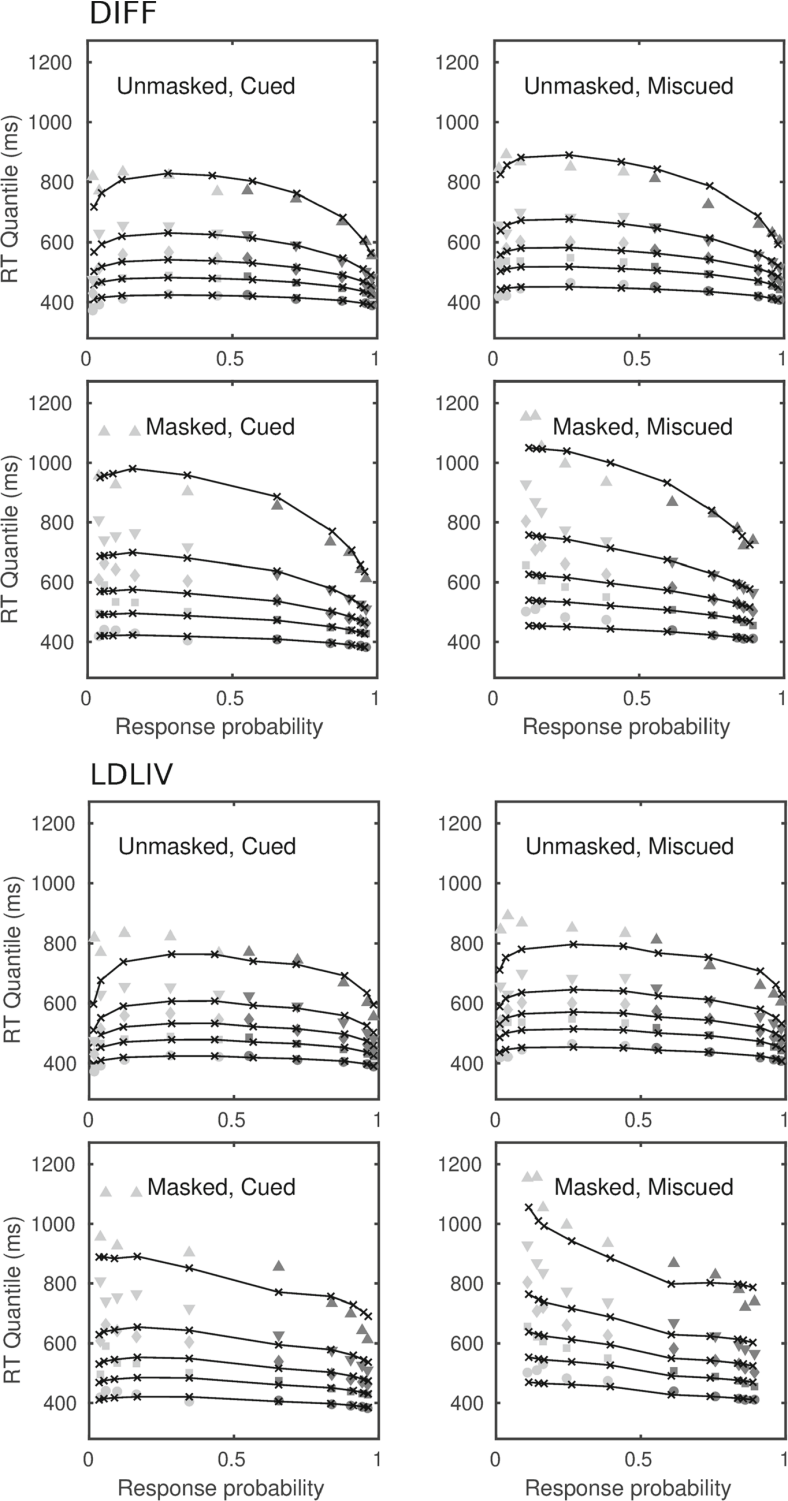
Quantile probability functions for responses in the Cue × Mask conditions for the diffusion model, DIFF, and the LDLIV model fitted to the Smith et al., (2004) spatial cuing data. The interpretation of the figure is the same as for Fig. 3

**Fig. 6:**
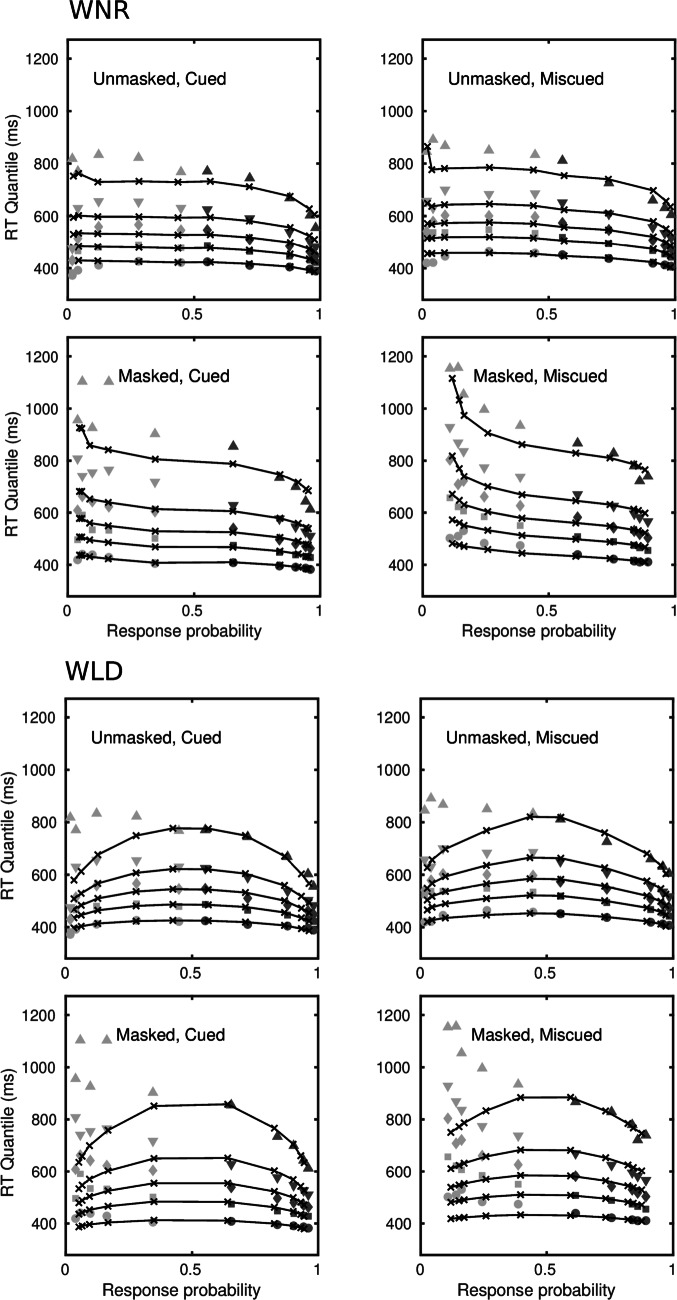
Quantile probability functions for responses in the Cue × Mask conditions for the reflecting Wiener model, WNR, and the racing Walds model, WLD, fitted to the Smith et al., (2004) spatial cuing data. The interpretation of the figure is the same as for Fig. 3

My treatment of the Smith et al., (2004) spatial cuing study follows the one in Smith and Ratcliff (2022), in which a separate model, with different drift rates, decision criteria, and nondecision times, was fitted to each cell in the Cue × Mask design for each participant. Smith et al., (2004) characterized the relationships among the conditions using an attention orienting model, which predicted how drift rates and nondecision times would change across the four conditions, but here I imposed no a priori theoretical constraints on the parameters because my primary concern was to compare decision models, not to test the attention model. Because there was no speed versus accuracy manipulation in the study, the starting point variability parameters could be omitted without worsening the fit and because the speed and accuracy of decisions to vertical and horizontal gratings were sufficiently similar they could be pooled to obtain a single distribution of correct responses and a single distribution of errors for each stimulus condition. This symmetry implies a symmetry constraint on the starting point in the diffusion model, *z* = *a*/2, and equality of the decision criteria in the dual-diffusion models, *a*_1_ = *a*_2_. I fit the same four models as for the Ratcliff et al. 2008 numerosity study. The averaged fit statistics are shown in Table 5; the comparative performance for the individual participants is shown in Table 6, and the estimated parameters are shown in Table 7. Figure 5 shows the performance of the diffusion and LDLIV models; Fig. 6 shows the performance of the reflecting Wiener and racing Walds models. I fit the models in the same way as for the Ratcliff et al. study, except I fit the models for each cell of the Cue × Mask design separately (24 fits in all). Smith and Ratcliff (2022) reported fits to the data from the Smith et al. study summed across the four cells of the design for each of the six participants, but as I have fitted independent models to each cell of the design I report them instead as 24 separate model fits, which better reflects the way they were carried out. Whether or not fit statistics are summed across cells for each participant before comparing the models has no effect on the inferences I wish to draw about them.

**Table 5:** Model fits for spatial cuing study

Model	*G*^2^	*k*	*df*	AIC	BIC
DIFF	122.2	9	46	140.2	176.1
LDLIV	124.0	13	42	150.0	201.9
WNR	136.7	12	43	160.7	208.6
WLD	179.9	13	42	205.9	257.8

**Table 6:** Comparative fits for the spatial cuing study by AIC and BIC

	#AIC				#BIC			
Model	DIFF	LDLIV	WNR	WLD	DIFF	LDLIV	WNR	WLD
DIFF	−	11	11	20	−	19	17	21
LDLIV		−	18	21		−	13	21
WNR			−	17			−	18

Number for which the row model was better than the column model (out of 24)

**Table 7:** Parameters for the spatial cuing study

DIFF	*a*	*ν*_1_	*ν*_2_	*ν*_3_	*ν*_4_	*ν*_5_	*η*	*T*_er_	*s*_*t*_		
	0.113	0.067	0.206	0.337	0.429	0.504	0.184	0.380	0.136		
LDLIV	*a*	*π*_1_	*π*_2_	*π*_3_	*π*_4_	*π*_5_	*ν*_sum_	*z*	*p*		
	0.382	0.551	0.642	0.745	0.822	0.887	2.851	0.036	4.078		
	*η*_1_	*η*_2_	*T*_er_	*s*_*t*_							
	0.192	0.003	0.292	0.089							
WNR	*a*	*π*_1_	*π*_2_	*π*_3_	*π*_4_	*π*_5_	*ν*_sum_	*r*			
	1.115	0.566	0.677	0.807	0.910	0.968	5.780	− 0.375			
	*η*_1_	*η*_2_	*T*_er_	*s*_*t*_							
	0.364	0.388	0.275	0.086							
WLD	*a*	*ν*_1_	*ν*_2_	*ν*_3_	*ν*_4_	*ν*_5_	*ν*_6_	*ν*_7_	*ν*_8_	*ν*_9_	*ν*_10_
	1.018	2.473	3.122	3.868	4.406	4.832	1.900	1.414	0.881	0.526	0.266
	*T*_er_	*s*_*t*_									
	0.260	0.072

The performance of the models on the spatial cuing task was similar to the numerosity task, except that the order of the diffusion model and the LDLIV model was reversed. The diffusion model was the best model, on average by *G*^2^, the AIC, and the BIC, followed by the LDLIV model, then the reflecting Wiener model, followed by the racing Walds model. The average *G*^2^ for the diffusion model was only marginally smaller than for the LDLIV model, but the average AIC and BIC were both considerably smaller because of the diffusion model’s greater parsimony. At the level of the individual fits, the picture differed, depending on the whether the AIC or BIC was used. By the AIC, the performance of the LDLIV model was slightly better than that of the diffusion model (13/24), but the BIC overwhelmingly favored the diffusion model (19/24). Some researchers have reported that the BIC has poorer model recovery properties than the AIC because of its bias against complexity (Donkin, Nosofsky, Gold, & Shiffrin, 2015; Oberauer & Lin, 2017; van den Berg, Awh, & Ma, 2014), but the picture that emerges if performance on both criteria is taken into account is similar to the one obtained from the Ratcliff (2008) study, which suggests that both the diffusion and LDLIV models provide good accounts of the data. The quantile probability plots in Fig. 5 further reinforce the picture obtained from the fit statistics. Both models captured the essential structure of the data fairly well, although there are differences in the way they characterize the slow error pattern, particularly in the two masked conditions, in which the slow error pattern is most pronounced. Whereas the .9 quantile functions for the diffusion model are uniformly concave for all conditions, the corresponding functions for the LDLIV model show a tendency to become convex. These qualitative differences in the predictions reflect differences in the way in which the models parameterize drift rate variability. Both models predict slow errors via drift rate variability: In the diffusion model, drift rates are normally distributed and may take on both positive and negative values; in the LDLIV model, drift rates are lognormally distributed and constrained to be positive. The differences in the .9 quantile functions in Fig. 5 are a reflection of this difference.

The qualitative pattern of predictions for the reflecting Wiener model in Fig. 6 resemble those for the LDLIV model in Fig. 5 but the effects are magnified. In the masked conditions, the quantile probability functions are concave for correct responses and convex for errors, which may arguably capture the qualitative structure slightly better than the other models, although the quantitative fit is worse. The model is appreciably worse than the LDLIV model by the AIC (6/24) but only slightly worse by the BIC (11/24) because the LDLIV model has an additional decay parameter that is heavily penalized by the BIC. As in the fit to the Ratcliff (2008) data, the reflecting boundary was estimated to be quite far from the starting point (more than a third of the distance of the starting point from the decision boundary), so I refit the model with *r* = − 1.5*a*, as I did previously. The mean *G*^2^ for the constrained model was 140.1 with associated AIC and BIC of 162.7 and 206.6, respectively. The AIC selected the model with the reflecting boundary as the better model; the BIC selected the model without it, again, because the BIC penalizes free parameters more severely. At the individual participant level, the model with the freely-varying reflecting boundary is preferred for only 9 of the 24 data sets by the AIC and 5 by the BIC. Like the fits of the model to the Ratcliff (2008) data, then, these fit statistics show the performance of the model is only marginally improved by the presence of a reflecting boundary.

**Fig. 7:**
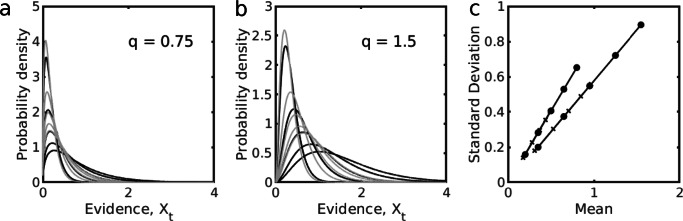
Distributions of evidence states in the accumulators in the LDLIV model, as described by Eq. 13. Figure 7a and b show distributions of evidence for *q* = 0.75 and *q* = 1.5, respectively. The black lines are for decay *p* = 0 and the gray lines are for decay *p* = 1.0. The five distributions in the figures are, left to right, for time *t* = 0.2, 0.4, 0.6, 0.8, and 1.0 s. Figure 7c shows *SD*(*X*_*t*_)/*M*(*X*_*t*_), the ratio of the standard deviation to the mean of the evidence distributions at each of the five time points. The steeper function is for *q* = 0.75 and the shallower function is for *q* = 1.5. In both instances the functions are linear. The filled circles are values for *p* = 0 and the crosses are for *p* = 1.0

Like the Ratcliff (2008) numerosity study, the quantile probability plots for the racing Walds model are almost symmetrical across the vertical midline, indicating the model is predicting essentially the same RT distributions for correct responses and errors. This failure to capture the structure of the distribution data is mirrored in the poor fits statistics for the model relative to the other models in Tables 5 and 6. Unlike the fits for the Ratcliff study, the model was not constrained by selective influence assumptions that held drift rates constant across speed and accuracy instructions. Instead, separate drift rates were estimated for each cell of the Cue × Mask design. The fits in Fig. 6 show that even without the selective influence constraint the model does not capture the relationship between the RT distributions for correct responses and errors across the full range of stimulus discriminabilities.

In sum, there is a high degree of agreement in the pictures that emerge from the fits to the Ratcliff (2008) numerosity study and the Smith et al., (2004) spatial-cuing study. The diffusion model performed well on both data sets, as has previously been shown, both by Smith and Ratcliff (2022) and in the original articles. The LDLIV model, which is new, also performed well, although it is less parsimonious than the diffusion model. This comparative lack of parsimony, as I commented previously, is a feature of dual-diffusion models, which require more free drift rate parameters than do the corresponding single-process model to characterize any given set of data. The reflecting Wiener model performed more poorly than the LDLIV model because it lacks a decay term to offset the tendency of dual-diffusion models to predict RT distributions that are more symmetrical than are found empirically. There was little evidence that the reflecting boundary contributed significantly to the performance of the model, which made it virtually identical to the racing Walds model, except for the inclusion of drift rate variability which the latter model did not have. The racing Walds model was consistently the worst model across both experiments, even though its number of free drift rate parameters was almost double those in the other models. The consistently poor performance of the racing Walds model highlights the importance of drift rate variability in accounting for the fine-grained properties of RT distributions and associated choice probabilities. As well as being theoretically principled, drift-rate variability is empirically necessary to provide satisfactory accounts of data.

### Distributions of evidence in the accumulators and drift rates across trials

Like the standard diffusion model, the LDLIV model accounts for experimental data using a combination of within-trial variability in evidence and across-trial variability in drift rates. As I proposed in the Introduction and develop in the Discussion, drift-rate variability can be viewed as an expression of the way in which the brain solves von Neumann’s problem. Unlike the standard diffusion model, neither the distributions of evidence in the accumulators nor the distributions of drift rates in the LDLIV model are normally distributed because of the positivity constraint on the LDLIV process in Eq. 2 and on the drift rates in Eq. 9. In this section, I characterize the distributions of evidence in the accumulators in the LDLIV model and show how they evolve over time. I also characterize the fitted distributions of drift rates used to model the experimental data.

The free transition density for an LDLIV process governed by Eq. 2 has the form (Giorno et al., 1989a, Eq. 6.2),

13 $$ \begin{array}{@{}rcl@{}} f(x, t | z, \tau) & = & \frac{p}{\sigma\left[e^{p(t - \tau)} - 1\right]} \exp\left\{-\frac{p\left[x + z e^{p(t - \tau)}\right]}{\sigma\left[e^{p(t - \tau)} - 1\right]}\right\} \\ & \times & \left[\frac{xe^{-p(t - \tau)}}{z}\right]^{(q - \sigma) / 2\sigma} I_{q/\sigma - 1}\left\{\frac{2p\sqrt{xz e^{p(t - \tau)}}}{\sigma\left[e^{p(t - \tau)} - 1\right]}\right\}. \end{array} $$

Figure 7a and b show examples of how the distribution of evidence in an accumulator grows as a function of time, as predicted by Eq. 13. These distributions are distributions of evidence states for a process unconstrained by absorbing boundaries; they are not distributions of nonterminated states. (Put otherwise, they are distributions of evidence when decision criteria are large and the probability of terminating before *t* = 1.0 s, the maximum time represented in the figure, is small). The functions show distributions of evidence for different values of decay, *p*, and the stimulus-dependent component of drift rate, *q*. As expected, the means and standard deviations both increase with the passage of time and the distributions become progressively less skewed. The rate of increase is faster for larger values of *q* and smaller values of *p*, again as expected. Empirically, the ratio of standard deviation to mean, *SD*(*X*_*t*_)/*M*(*X*_*t*_), follows a simple linear, Weber’s Law like, scaling relationship, which is identical for large and small values of decay, *p*. I computed the mean and standard deviations in the figure numerically from the density function in Eq. 13 because there do not appear to be closed-form expressions for the moments. The slope of the line relating the standard deviation to the mean is steeper for smaller values of *q* because when *q* is small the mean grows more slowly relative to the standard deviation.

If we would like to interpret the diffusion process in the LDLIV model as an approximation to the neural processes of evidence accumulation, then the distributions in Fig. 7 have the right kinds of general properties. The distributions of evidence are positively-valued and positively-skewed, and their means and standard deviations both increase with time, as we would expect from neural firing rates (e.g., Roitman and Shadlen (2002) Fig. 4). The statistics of neural spike trains are often characterized using the so-called “Fano factor,” defined as Var(*X*_*t*_)/*M*(*X*_*t*_), that is, as the ratio of the variance of the firing rate to its mean (Rieke, Warland, de Ruyter van Steveninck, & Bailek, 1999, p. 52). In an idealized model neuron in which the firing rate is a Poisson process the Fano factor is unity, but, as Reike et al. point out, Fano factors for real neurons are highly variable and may be much less than or much greater than the Poisson ideal and may also vary systematically with time. Any identification of the properties of the LDLIV model with the underlying neural dynamics would most plausibly be made at the level of the neural populations rather than the individual neurons, so we should not necessarily expect distributions of evidence in the accumulators to mirror the firing rates of individual neurons because they are at different levels of description.

**Fig. 8:**
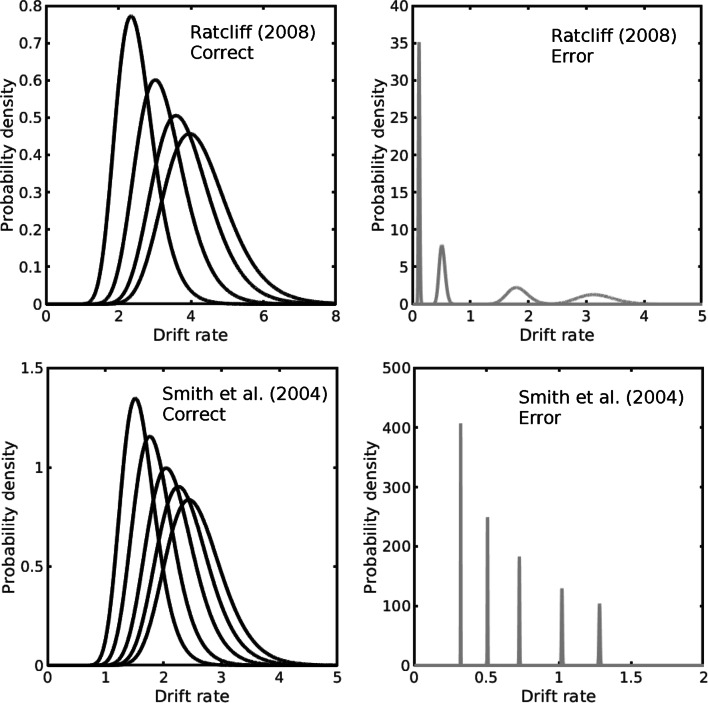
Distributions of drift rates for the LDLIV model in the correct response and error accumulators for the Ratcliff (2008) study (upper panels) and the Smith et al., (2004) study (lower panels). The distributions are lognormal distributions parameterized as in Eq. 9 with parameters given by Tables 4 and 7, respectively

Figure 8 shows the fitted distributions of drift rates for correct response and error accumulators in the LDLIV model for the Ratcliff (2008) and Smith et al., (2004) studies. The distributions in the figure are lognormal distributions parameterized according to Eq. 9 using the averaged parameters in Tables 4 and 7. For correct response accumulators, the distributions are for increasing stimulus discriminability (increasing *π*_*i*_*ν*_sum_) left to right. For error accumulators, the distributions are for decreasing stimulus discriminability (decreasing (1 − *π*_*i*_)*ν*_sum_) left to right. For both studies, the drift rate variability in the error accumulators was much less than in the correct response accumulators. Indeed, in the Smith et al. study the variability in the error drift rate was negligible and the distributions approach Dirac delta functions. Although the drift rate distributions are not algebraically normal, they are nevertheless fairly symmetrical, as we would expect if drift rates arise by summing or averaging independent noisy elements in a population. If so, we would expect the distributions of drift rates to approach a limiting normal form but not necessarily to fully achieve it, as I discuss subsequently.

## Discussion

I have used von Neumann’s (1956) problem of how to build reliable organisms from unreliable components as a point of departure to motivate a new model, the LDLIV model, which embodies three neurally-inspired elements of model construction that the standard diffusion model lacks, namely, positivity, boundedness, and separate evidence totals for different responses. The resulting model performed similarly to the diffusion model in the fits to the data from two studies — somewhat better than the diffusion model for one, similarly or somewhat worse than the diffusion model for the other, depending on the model selection criterion — and systematically better than two other dual-diffusion models that embodied some but not all of these elements. To the extent that these elements are deemed important for model construction the performance of the LDLIV model is reassuring, because it shows that a mathematically tractable, neurally-inspired, diffusion model yields similar empirical predictions to the original model.

I chose to use von Neumann’s problem to motivate the LDLIV model for two reasons. First, it highlights the unity and continuity of a body of theory that extends back to Fechner (1860) and forward into the twentieth century through Thurstone’s (1927) law of comparative judgment and signal detection theory (Green and Swets, 1966) to the sequential-sampling models of the 1960s and 1970s. As I noted in the Introduction, Link (1994) argued that Fechner had a well-articulated theory of decision-making based on Gauss’s theory of errors, which holds that probabilistic, normally-distributed variation in measurement will arise when the measurements are aggregates of elements, each of which is independently subject to error. The modern expression of this result is the central limit theorem (Breiman, 1968, ch. 9) and its particular power comes from the fact that the distribution of the aggregate is predicted to be normal, regardless of the distribution of the errors.^6^ In choosing Gauss’s theory to characterize the processes underlying the variability of psychophysical decisions, Fechner thus appears to have understood that the brain solves von Neumann’s problem in the way von Neumann proposed, by aggregation. The second reason for using von Neumann’s problem is that it highlights an essential philosophical difference between models that seek to provide a solution to his problem and those that do not. The latter include a miscellany of models that represent evidence accumulation as a deterministic rather than a stochastic process. These include the Grice model (Dzhafarov, 1993; Grice, 1972), the “random ray” model (Reeves, Santhi, & DeCaro, 2005), the LATER model (Carpenter, 2004), and the ballistic and linear ballistic accumulator (LBA) models (Brown & Heathcote, 2005; 2008).

### Deterministic versus stochastic evidence accumulation

Recent treatments of decision processes have sometimes used the term “evidence accumulation model,” as a synonym for sequential-sampling models, as I have defined them here, as models in which evidence is accumulated by summing successive samples from a noisy evidence sequence. However, the term is often used more inclusively, to encompass the deterministic growth models in the preceding paragraph. Although such models are often referred to as evidence accumulation models, strictly speaking they are not — or not in the sense in which “evidence accumulation” is used in relation to sequential-sampling models. Rather, all of the evidence used to make a decision in such models, in the form of a probabilistic representation of the discriminative information in the stimulus, is present at the beginning of the trial, or, more precisely, by the end of the stimulus encoding part of the nondecision time. In so-called piecewise ballistic accumulation (Holmes, Trueblood, & Heathcote, 2016), the encoded evidence state can change if the physical stimulus changes but in either version of the model there is no accumulation of evidence comparable to that which occurs in sequential-sampling models. Instead there is deterministic growth of an activation state that translates the evidence encoded at the beginning of the trial — or as recoded at discrete change points — into an overt response. The decision time component of RT is the time needed to effect this translation.

Deterministic growth models like the LBA (in either its original or piecewise form) were not designed to provide a solution to von Neumann’s problem. Rather, these models have the pragmatic aim of providing a so-called “measurement model” that allows meaningful components of processing, like decision criteria, rate of evidence growth, or nondecision time, to be estimated from data. Much of the attraction of a model like the LBA is its comparative simplicity and the ease with which such components can be estimated (Brown & Heathcote, 2008). Although this is a defensible objective, it does beg the question, if the model is not conceptualized as being a model of what the brain is actually doing, then what criteria can be used to validate the resulting estimates? Sometimes the LBA has been validated by pointing to the similarity between its parameter estimates and those of the diffusion model (Donkin et al., 2011), but this strategy relies on the existence of a model that can be used as a gold standard against which other models can be validated. But if the diffusion model is deemed to be the gold standard, why not simply use it? The LBA has also been validated by showing that its estimated parameters behave in predictable and interpretable ways in response to experimental manipulations (Dutilh et al., 2019). Such demonstrations are important, but they provide little help in establishing what, if any, are the limits of this kind of interpretability.

In contrast to the pragmatic spirit that motivated the development of models like the LBA, sequential-sampling models like the diffusion models seek to solve a fundamental and well-posed theoretical problem, which I have characterized as von Neumann’s problem in time: If the elements that encode cognitive representations are subject to moment-to-moment noise, as neural recordings from decision-related brain structures suggest to be the case, then how can reliable decisions be made based on those elements? The models provide a simple, compelling, and theoretically-satisfying answer to this question: by temporal integration. The speed-accuracy tradeoff, which is one of the most basic and ubiquitous phenomena in decision making (Luce, 1986, ch. 6.5), follows immediately from the assumptions of these models: The more evidence that is accumulated, the more reliable the resulting decision will be.

Unlike sequential-sampling models, deterministic growth models have a problem in explaining why errors occur at all. The LBA assumes that errors occur because of variability in drift rates. As a result, evidence sometimes grows more rapidly in the error accumulator than in the correct response accumulator, leading to an error. But this does not suffice to explain the speed-accuracy tradeoff: If evidence growth is deterministic, then accuracy after a long period of growth will be the same as after a short period, so changing decision boundaries in itself buys you nothing. The LBA’s solution to this problem is to assume that longer periods of evidence accumulation help overcome starting point biases. If the starting points for evidence accumulation are biased towards the wrong response, then increasing decision boundaries will help to offset the bias and accuracy will be greater with large than with small boundaries.

In a purely technical sense, this explanation works. The LBA has been fitted to a number of data sets in which discriminability has been crossed with speed versus accuracy instructions and has captured the resulting data successfully. It is therefore unlikely we could expect to adjudicate between deterministic and stochastic models on the basis of fit statistics alone. A more relevant question to ask is: what is being explained in each case? In diffusion models, the speed-accuracy tradeoff is an expression of the model’s solution of von Neumann’s problem, which is a deep and general problem about the reliability of biological computation and of how neurons transmit information statistically. In the LBA, the speed-accuracy tradeoff is an expression of how starting-point bias interacts with the rates of evidence growth. These biases were introduced into the design of the model because without them it would be unable to predict speed-accuracy tradeoffs. In diffusion models, the speed-accuracy tradeoff predictions are an expression of a solution to a general theoretical problem; in the LBA model, they are an expression of a feature of the model that was put there for the express purpose of explaining why speed-accuracy trade-offs occur.

A possible response to the validation question for deterministic models is to argue that a model like the LBA or the Grice model *is* a model of what the brain is doing. The argument is sometimes made that if the population of neurons that encode cognitive representations is large then the central limit theorem will ensure that the functions that characterize evidence accumulation in the population will be sufficiently close to deterministic that they can characterized satisfactorily by deterministic dynamics like those in the LBA and the Grice model. Although this position is arguable, there are two obvious objections to it. The first is that the neurons that encode a stimulus in a population are not independent but weakly-coupled and in weakly-coupled networks the usual central-limiting arguments do not apply. Such networks can continue to exhibit large variability even when the number of neurons in the network becomes large (Bair et al., 2001, Mazurek et al. (2003) Zohary, Shadlen, & Newsome, 1994), so near-deterministic dynamics is not an inevitable consequence of large populations. A second objection — setting aside the properties of weak coupling — is why, if the populations are sufficiently large that the moment-to-moment stochasticity in evidence accumulation is almost entirely averaged out, there is still large trial-to-trial variability in the rates of evidence accumulation in the LBA or in decision criteria in the Grice model? If the averaging is sufficient to remove moment-to-moment variability, why does significant trial-to-trial variability remain? While it may be possible to argue that within-trial and across-trial variability have different sources, one of which averages out and one of which does not, the theoretical argument for why significant, residual across-trial variability should remain in the process after all of the within-trial variability is averaged out has not been articulated.

### Drift rate variability

In the Introduction I emphasized the close theoretical relationship between sequential-sampling models and earlier models of psychophysical decision-making like signal detection theory that assume normally-distributed stimulus representations. I characterized those models as solving von Neumann’s problem in space — in the sense of aggregating information across members of a neural population, or more abstractly, across a set of noisy encoding elements — and the sequential-sampling models as solving his problem in time. Viewed in this way, there is a close relationship between the assumption that evidence is accumulated by a diffusion process and the assumption that the evidence entering the process is normally distributed across trials. Both of these assumptions follow from the larger theoretical argument that the primary impediment to reliable computation in the brain and central nervous system is the need to solve von Neumann’s problem. This view of the complementarity of diffusive evidence accumulation and normally-distributed drift rates contrasts with recent discussions that have portrayed the drift-rate assumptions as arbitrary and ad hoc. Jones and Dzhafarov (2014, p. 24) asserted: “One possibility is that the Gaussian distribution is psychologically correct, but this is doubtful for three reasons. First, there is no clear reason to expect a Gaussian. Whereas the Gaussian distribution of within-trial variability emerging from a diffusion process can be explained by the summation of many independent neural events (via the central limit theorem), there is no obvious candidate for a between-trial analog—that is, a large number of independent and identically distributed between-trial variables that sum to determine the drift rate.” Heathcote, Wagenmakers, and Brown (2014, p. 677) similarly remarked: “It is true that the particular forms of the across-trial variability parameters in decision-making models (Gaussian and uniform) were originally chosen arbitrarily, for practical and not theoretical reasons.” In a similar vein, Tillman et al. (2020, p. 913) argued: “...the between-trial variability assumptions are not part of the process model that explains how the RT data are generated. The core process of sequential sampling models is the integration of evidence to a threshold, which explains how someone makes a decision, but the between-trial variability assumptions provide no additional explanation of the decision process and are simply added to help the models fit data.”

Contrary to these portrayals of drift-rate variability as theoretically unmotivated, I view it as a natural expression of how the brain solves von Neumann’s problem, namely, by aggregating across a population of elements that each provide a noisy representation of the information in the stimulus. The strongest objection I can see to the argument that drift rates will be normally distributed is the work Zohary et al., (1994) and others implying that neurons in a population are weakly-correlated rather than independent, which means that a simple central-limit theorem argument may not apply. If the elements in a representation are correlated, then its quality may not improve in proportion to the square root of the number of elements that compose it as implied by the central-limit theorem and, indeed, depending on the correlations in the population, the result need not be normally distributed. However, in the absence of some specific, neurally-derived model of drift-rate variability, the normal distribution remains the most natural one to assume on general theoretical grounds as an expression of the aggregation of elements within a population.

If normal distributions of drift rates indeed arise because they are aggregates across populations of independent, noisy elements, then this might seem to be a reason to prefer the standard diffusion model over the dual diffusion models, in which the drift rates are nonnormal. However, it should be remembered that the normal distribution is the limiting form of the distribution when the number of noisy elements contributing to it is large and the limiting form may be more or less well approximated depending on the size of the population. In the case of the LDLIV model, those elements, whether they are the firing rates in individual neurons or some more abstract entities like the “evidence samples” in the sample size model (Smith, Corbett, Lilburn, & Kyllingsbæk, 2018), are constrained to be positive by virtue of the diffusion equation, Eq. 2. Sums of even highly-skewed, positively-valued random variables converge to the normal distribution in the limit — the sum of single degree-of-freedom chi-square random variables is a well-known example — so the positivity of the elements does not preclude a normal probability law for the sum or the average in the limit. The drift rate distributions in Fig. 7, although not algebraically normal, are fairly symmetrical, as we would expect if they approach a limiting normal form but do not fully achieve it. The fact that the model does not assume normal distributions of drift rates does not invalidate the broader argument that drift rate variability is a reflection of the way in which the brain solves von Neumann’s problem.

### Competitive interactions, continuous motor processes, and model mimicry

I motivated the LDLIV model via three neurally-inspired design principles — separate accumulation, positivity, and boundedness — and showed that a model embodying these principles performs comparably to the standard diffusion model in accounting for empirical data. However, there are other neurally-inspired design principles that researchers have argued are important. One is competitive interaction or mutual inhibition between accumulating evidence totals; another is continuous flow of activation from the decision process to the motor system. It is beyond the scope of this article to evaluate either of these principles systematically, but arguments have been made for the importance of both of them, so I discuss them briefly below.

Competitive interaction among representations is a powerful computational principle that can implement, among other things, efficient selection of targets from among distractors in search tasks (Smith & Sewell, 2013; Smith, Sewell, & Lilburn, 2015). Competitive interactions implemented by multiplicative, or “shunting,” differential equations lead to divisive normalization models in which the strength of activation in a representational unit is divided by the sum of the strengths of the units with which it competes (Grossberg, 1980). Carandini and Heeger (2012) reviewed evidence suggesting that divisive normalization is ubiquitous in the brain. Grossberg (1980) argued that divisive normalization allows the brain to retain its sensitivity to small stimulus differences without saturation under conditions in which stimulus intensities may vary over several orders of magnitude. For decision modelers, however, the question of interest is not the general one of whether or not these kinds of competitive interactions occur neurally but the narrower one of whether they occur between accumulating evidence states in a sequential-sampling decision model.

The prototypical competitive interaction model is the leaky competing accumulator (LCA) model of Usher and McClelland (2001), which represents the decision process as a race between mutually-inhibitory OU diffusion processes constrained to be positive by reflecting boundaries. Ratcliff and Smith (2004) found that an LCA model with lateral inhibition performed no better than a dual diffusion model with drift rate variability and decay but no lateral inhibition. Purcell, Schall, Logan, and Palmeri (2012) used an *n*-alternative version of an LCA model to model visual search for a target stimulus among distractors in monkeys performing a saccade-to-target decision task. They found that RTs were better described by a model, which they called a “gated accumulator model,” which had competitive interactions among accumulators, than they were by a model without such interactions. Cox, Palmeri, Logan, Smith, and Schall (2022) combined the competitive interaction model of attentional selection of Smith and Sewell (2013) with the gated accumulator model of Purcell et al. to jointly model firing rates in frontal eye field neurons and distributions of RT in a saccade-to-target decision task. A novel feature of Cox et al.’s approach was that they were able to characterize the importance of different computational mechanisms, such as recurrence, feedforward inhibition, and lateral inhibition, to the firing rates of the individual neurons in their sample. When analyzed in this way, there were a subset of neurons whose activity was best modeled by assuming lateral inhibition, although its contribution to overall fit was less important than recurrence and feedforward inhibition.

The evidence for lateral inhibition in search is unsurprising because successful search requires resolution of the competition between targets and distractors and lateral inhibition is an effective way to accomplish this, especially when it is nonlinear (Cox et al., 2022; Grossberg, 1980; Smith & Sewell, 2013; Smith et al., 2015). However, an identified role for lateral inhibition in search leaves open the question of its role in tasks not involving inhibition or suppression of distractor stimuli. Usher and McClelland (2001) used lateral inhibition in their LCA model as an alternative to drift-rate variability as a way to predict slow errors.

Lateral inhibition in race-model architectures has recently been implicated in the absolute-intensity or absolute-value effect (Kirkpatrick et al., 2021; Ratcliff, Voskuilen, & Teodorescu, 2018; Teodorescu & Usher, 2013; Teodorescu, Moran, & Usher, 2016). This is the finding that decisions about differences between pairs of stimuli of equivalent intensity, strength, or value are faster if they are stronger than if they are weaker. Teodorescu and Usher and Teodorescu et al. argued that these findings are better characterized by a racing-processes decision architecture than by a single-process architecture and found the best model was the LCA model, which has inhibition between accumulating evidence states. Ratcliff et al. subsequently showed that a single-process diffusion model in which drift-rate variability scales with stimulus strength can predict the absolute-strength phenomena reported by Teodorescu and colleagues. Kirkpatrick et al. fit several versions of the LCA model, including models without leakage and without inhibition, to data from two absolute-intensity tasks and found the full LCA model performed better than any of the reduced versions of it. This result in itself is unsurprising because, as shown by Smith and Ratcliff (2009), leakage and drift-rate variability are both needed to capture the shapes of RT distributions for correct responses and errors in racing-accumulator architectures and, in the absence of drift-rate variability, inhibition allows models to predict slow errors. However, Kirkpatrick et al. also showed that the full LCA model augmented with the same extra sources of variability used by Ratcliff et al. still perform better than any of reduced versions of it for a majority of participants. Their results suggest that, in these kinds of paradigms at least, inhibition is doing something more than drift-rate variability alone. This finding is suggestive, but it was based on very small samples (48 trials/condition) and the graphical presentation of fit did not include any representation of distribution shape or the relationship between correct responses and errors. Moreover, even at the level of the choice probabilities and mean RTs the authors used to assess fit qualitatively the best LCA model showed misfits in some conditions. Clarifying these relationships, using models like the LDLIV model, will require further research.

Another open question concerns the relationship between the decision process and the motor process it drives. There are two parts to this question. The first concerns the additive decomposition of RT into a sum of independent random variables representing decision and nondecision processes (the latter designated *T*_er_ in the models considered here). The second concerns the form of the distribution assumed for *T*_er_. The additive decomposition is an expression of the stage-dependent processing assumption formalized in Sternberg’s (1969) additive-factors method: RT is the sum of the times required to complete a sequence of independent processing stages and processing in a stage only begins once the previous stage is complete. If the decision process is viewed as a discrete stage in an additive-factors architecture then completion of the decision stage is a point event that can be identified theoretically with the process reaching a decision criterion or boundary.

From a neural modeling perspective, boundary crossings are idealized, abstract events, and models in which there a continuous flow of activation from the decision process to the motor system may seem intrinsically more plausible, given the highly interconnected nature of the brain as a whole. Researchers like Kelly, Corbett, and O’Connell (2021), for example, have argued that we should conceptualize evidence accumulation as being essentially a motor preparation process. In this spirit, Verdonck, Loossens, and Philiastides (2021) proposed a continuous-flow motor activation model in which a Wiener diffusion process drives a leaky integrating threshold (LIT) process. In the LIT model, activation in the motor system, *Y*_*t*_, is related to accumulated evidence in the decision process, *X*_*t*_, by the linear differential equation

14 $$ dY_{t} = (\beta X_{t} - \lambda Y_{t}) dt. $$

In this equation *β* is a scaling or gain parameter and *λ* is a motor system decay parameter. In comparison to the Wiener process model, the integrated evidence in the LIT model is subject to an additional integration, with decay, in order to make a response. This occurs when the doubly-integrated process reaches a threshold. Verdonck et al. derived explicit expressions for the statistics of the LIT process *Y*_*t*_ in Eq. 14 and compared the model to the standard diffusion model on three different data sets. To do so, they used the *D* ∗ *M* (i.e., decision convolved with motor time) approach of Verdonck and Tuerlinckx (2015) to estimate the distribution of the motor time empirically and found the LIT model was the best-fitting model in each instance.

A striking and unexpected feature of the LIT model — not remarked on by Verdonck et al., (2021) — is that the motor activation process *Y*_*t*_ is identical to the integrated OU process in the Poisson shot-noise model of Smith (2010), described earlier. This equivalence is surprising, given the divergent semantics of the models. In Smith’s model, the instantaneous state of evidence entering the decision process is represented by the difference between a pair of Poisson shot-noise processes that describe the flux in the excitatory and inhibitory postsynaptic potentials induced by a sequence of action potentials. The instantaneous evidence is accumulated over time to make a decision (in either a two-boundary or a racing-process architecture). At high neural firing rates, represented by high-intensity Poisson processes, the shot-noise difference process approximates an OU process and the accumulated evidence, obtained by integrating the instantaneous evidence state over time, approximates an integrated OU process, *U*_*t*_. The equivalence of the LIT and integrated OU models can most easily be seen by comparing Eqs. 19 and 20 in Smith (2010), which give the mean and variance of the integrated OU process, *U*_*t*_, with the unnumbered equations between Eqs. 5 and 6 in Verdonck et al., which give the mean and variance of the LIT process, *Y*_*t*_. Except for scaling constants, the two sets of equations are identical.

Equality of the mean and variance of two stochastic processes does not mean the processes themselves are identical, but in fact the motor activation process, *Y*_*t*_, in the LIT model and the integrated OU process, *U*_*t*_, in the shot noise model, are identical in the sense that they satisfy the same stochastic differential equation. Verdonck et al., (2021) showed that the process *dY*_*t*_/*dt* in the LIT model (the unnumbered equation beneath their Eq. 2) has a form that can be recognized as a linear functional representation of the OU process (Bhattacharya and Waymire, 1990, p. 581, Eq. 2.49). The integral of this process is the integrated OU process, *U*_*t*_, so it follows that *Y*_*t*_ and *U*_*t*_ satisfy the same stochastic differential equation. This being so, the question of where the boundary or threshold is placed theoretically may not be as consequential for a model’s performance as first appearances might suggest. The reader is referred to Smith (2010) for a characterization of the integrated OU process as a model of decision making and its relationship to the Wiener process, which it approaches asymptotically.

The second part of the motor-process question concerns the distribution of the nondecision time, *T*_er_. Many researchers (myself included), have followed Ratcliff and colleagues (e.g., Ratcliff and Tuerlinckx (2002)) and modeled *T*_er_ as uniformly distributed with range *s*_*t*_. The justification for using a uniform distribution is the pragmatic one that, if the variance of the nondecision time is small relative to that of the decision time, then the shape of the smaller component does not much matter, because the standard deviation and the shape of the predicted RT distribution will be almost completely determined by those of the decision time distribution. If so, then the uniform distribution, which is easy to handle computationally, is as good as any other. Some researchers have proposed alternatives to this approach, such as Smith (1989) who used distributions of simple (i.e., one-choice) RT as estimates of the nondecision time distribution and deconvolved them from the distributions of RT using Fourier methods (Smith, 1990) to obtain estimates of the distributions of decision times. However, this approach is open to the objection that the component being deconvolved out may be too large. Essentially, it relies on the assumption — first made by Donders (1869) and termed the “assumption of pure insertion” by Sternberg (1969) — that the duration of a processing stage can be estimated by comparing RTs on two different tasks that are thought to differ by the presence or absence of the stage in question. As emphasized by Sternberg, there is no way to verify this assumption and there may be good grounds to doubt it.

A novel approach to estimating the distributions of motor times was suggested by Verdonck and Tuerlinckx (2015), with their so-called *D* ∗ *M* method. They observed that a common, unobserved component of RT can be estimated from a pair of experimental conditions by cross-convolution. If *g*_1_(*t*) and *g*_2_(*t*) denote the decision-time density functions in two different conditions and *r*(*t*) is their common motor density, then the observed RT density functions, *f*_1_(*t*) and *f*_2_(*t*), will be the convolutions of the decision time and motor time densities, if the decision and motor times are independent random variables. In symbols, *f*_1_(*t*) = *g*_1_(*t*) ∗ *r*(*t*) and *f*_2_(*t*) = *g*_2_(*t*) ∗ *r*(*t*), where the asterisks denote convolution. Verdonck and Tuerlinckx pointed out that if the assumption of a common, independent motor component holds then the decision-time components can be estimated by computing the cross-convolution of the empirical RT distribution with the candidate decision time distribution for the *other* condition, that is, *f*_1_(*t*) ∗ *g*_2_(*t*) and *f*_2_(*t*) ∗ *g*_1_(*t*). If the candidates for *g*_1_(*t*) and *g*_2_(*t*) have been correctly specified then the two cross-convolutions should be equal to each other because *f*_1_(*t*) ∗ *g*_2_(*t*) = *f*_2_(*t*) ∗ *g*_1_(*t*) = *g*_1_(*t*) ∗ *g*_2_(*t*) ∗ *r*(*t*), in either instance. The best estimates of the decision time distributions will therefore be the ones that makes the difference between the two empirical cross-convolutions as small as possible. Once these components have been estimated the unknown motor component can be recovered, in principle at least, by deconvolution. To date the method has not been widely applied, except by its developers, and many researchers may wish to see more simulation and empirical studies before embracing it fully as a data-analytic tool. Nevertheless, the method appears to represent a welcome attack on a problem that for a long time has seemed intractable.

### Levels of description in decision models

I characterized the LDLIV model as “neurally-inspired,” in the sense that it embodies design features that neural decision-making studies have suggested are desirable in a cognitive model, namely, separate accumulation, positivity, and boundedness or saturation. I noted that diffusion model representations of the process of evidence accumulation can be obtained either in a top-down way, from mean-field models of attractor dynamics, or in a bottom-up way, from the statistics of the flux of postsynaptic potentials induced by a stream of action potentials. Diffusion processes therefore provide a natural way to represent the statistical properties of ensembles of neurons. Nevertheless, it has sometimes been argued these kinds of models, regardless of their architecture, lack neural realism, and that a truly “neural” explanation needs to be at a more granular level, as exemplified, for instance, by the spiking neuron model of Wang and colleagues (Lo & Wang, 2006; Wang, 2001; 2002; Wong & Wang, 2006). As noted previously, the Wang model is an attractor model composed of spiking neurons with temporal response properties that embody the properties of real neurons. The model was developed originally to try to solve the problem of how to bridge the gap between the different time scales of cognitive and neural integration. Cognitive models assume that evidence is integrated, or accumulated, on a time scale of around a second or so, but as Wang has argued, the longest neural integration times are around 50-100 ms, which is an order magnitude shorter than this, and the wrong time scale on which to implement evidence accumulation behaviorally. He proposed that behavioral-level integration is implemented by persistent activity in reverberation loops and his spiking neuron model embodies this idea in its architecture. The model captures the main features of choice probabilities and mean RTs but, until recently, it had not been shown to characterize RT distributions for correct responses and errors in the level of detail I have considered here.

Recent progress on this question was reported by Umakantha, Purcell, and Palmeri (2022) who fitted the diffusion model to simulated data from the Wang model and found that the choice probabilities and RT distributions for correct responses were well-fitted in all cases — although the quality of the fit to the RT distributions is difficult to infer from their figures because they plotted it using cumulative distribution functions (see my earlier remark on the use of cumulatives in depicting fit). Beyond questions of fit, Umakantha’s larger question was whether there was mutual translatability between the two models, that is, whether psychological parameters of the diffusion model had interpretable physiological correlates in the parameters of the Wang model, and vice versa. In some instances, parameters of the diffusion model like drift rate, boundary separation, and nondecision time had identifiable physiological analogues in the Wang model, but in other instances the mapping between the models was less clear.

Models like the Wang model raise a general philosophical question about the appropriate level of description at which to explain decision making or other cognitive phenomena. One view is that the more neural detail the better, and that a description at the level of a network of spiking neurons is more biophysically grounded and in some sense more scientifically “real” than a description at the level of a diffusion process. My view is that there are multiple levels of description on which we might seek to characterize a phenomenon like decision making, which differ in their granularity or resolution and that the best level of description is not necessarily the most granular one. Rather, it is the one at which the phenomenon appears simplest and most lawful. Nevertheless, a complete scientific explanation needs to characterize how the explanatory entities at one level of description are related to those at levels below it. Boden (1972) used the term “empirical reduction” to describe explanations of this kind, which she distinguished from “strict reduction.” In strict reduction, all statements about phenomena at a higher level of description are translated into statements at a lower level and are replaced by them. In empirical reduction, the explanatory entities used to explain phenomena at a higher level are characterized in terms of entities at lower levels. In Boden’s terms, the study by Umakantha et al., (2022) can be viewed as an attempt to seek an empirical reduction of the diffusion model to the Wang model.

From a somewhat different standpoint, Smith and McKenzie (2011) attempted to provide an empirical reduction of Wang’s idea that evidence is accumulated in recurrent loops and sought to show how it leads to diffusive evidence accumulation at a behavioral level. They combined the Poisson shot noise model of Smith (2010) with a model of the statistics of recurrent loops, in which each spike traveling around a loop initiates a new spike after an exponential decay and in which spikes entering the loop cumulate with those already present by superposition. They derived a limiting diffusion equation for the statistics of such loops, which has the form of an OU process with linearly increasing drift and diffusion rate, whose macro properties closely resemble those of the Wiener process. When combined with an appropriate stopping rule in a dual-diffusion architecture the model successfully predicted RT distributions for correct responses and errors and choice probabilities about as well as do the models I considered here. The point of Smith and McKenzie’s demonstration was not that the recurrent loop shot-noise model provides a better, or an alternative, model to existing cognitive models, but rather to show that recurrence, as a model for long-time-scale temporal integration, leads in a precise way to a representation of evidence accumulation as a diffusion process.

Researchers working on applied cognitive questions in which decision models are used, considering the results in this article, may ask what implications they have for their own practice and for the decision model they use to analyze their data. Should they prefer the LDLIV model over the standard diffusion model because it is more “neural” and hence more likely to approximate the scientific ground truth? My results imply that there is substantial mimicry between the two models and that in many settings it is likely they will provide similarly good accounts of empirical data. There is a high degree of convergence between the diffusion model, which is a purely cognitive model, and the LDLIV model, which is a cognitive model augmented with some additional neural design principles that the diffusion model lacks. Whether or not these additional design principles are embodied in a model does not appreciably change the quality of the fit at the individual participant level, but importantly, they do not make it worse. Arguably more important than the differences between the two models are their points of similarity. Both model represent the decision process as an accumulation of noisy, continuously-varying evidence by a diffusion process and both predict slow errors by across-trial variability in drift rate. Both models provide a solution to von Neumann’s problem, as I have defined it here, and moreover, both models solve it in a similar way, by a combination of within-trial and across-trial variability.

## Conclusion

In this article, I have introduced a new dual-diffusion model for decision making, the LDLIV motdel, which embodies three neurally-inspired principles of evidence accumulation: separate accumulation, boundedness, and positivity, and have shown that it accounts successfully for two benchmark sets of experimental data. Unlike previous models that have imposed a positivity constraint on the accumulating evidence on grounds of neural plausibility, the evidence in the LDLIV model is naturally restricted to the positive real line without artificial barriers like reflection or truncation. To the extent that its dynamics constrain it to the positive real line, the process can be viewed as a natural expression of the idea that the diffusion process is a cognitive-level expression of evidence accumulation processes implemented by neural firing rates. The distributions of evidence in the process are positively skewed, again as would be expected of processes implemented by neural firing rates, and obey a simple scaling law in which the mean and standard deviation grow in proportion to one another, independent of decay. Like other successful dual diffusion models, the model assumes that evidence decays while it is being accumulated, leading to saturation at high intensities. Also, like other successful single and dual diffusion models, the model accounts for slow errors via across-trial variability in drift rate. I emphasized in the Discussion that the construct of drift-rate variability in the models is neither ad hoc nor arbitrary, but, like the evidence accumulation process itself, is an expression of how the brain solves von Neumann’s problem of building reliable organisms from unreliable components.

## Figures and Tables

**Fig. 9:**
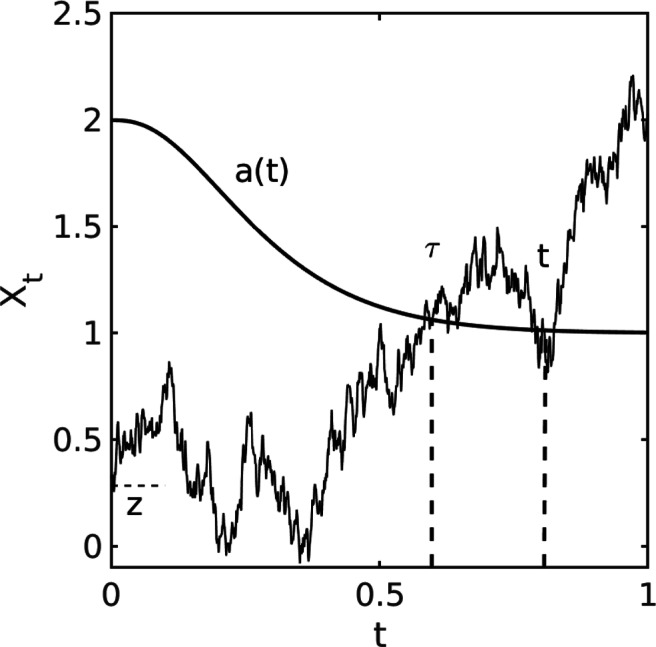
Fortet decomposition of the sample path of a diffusion process, *X*_*t*_, starting at *X*_0_ = *z*, through a time-varying decision boundary, *a*(*t*). Equation A1 gives a decomposition of *f*[*a*(*t*), *t*|*z*,0], the probability density associated with a point on the boundary, *a*(*t*), at time *t*. The time of the first boundary crossing is *τ*, *τ* ≤ *t*. After crossing at *a*(*τ*) the process then makes a transition to the point *a*(*t*) in time *t* − *τ*, possibly with an unspecified number of the further boundary crossings during this interval. In the figure the symbol *t* is used both to denote the time index of the process and to denote the distinguished point at which *X*_*t*_ = *a*(*t*)

## Data Availability

Data associated with this study are publicly available at https://osf.io/8na3f.

## Appendix A: Integral equation representation of the first-passage time distribution for a diffusion process

The basis of the integral equation method is a general decomposition, attributed by Durbin (1971) to Fortet (1943) and shown in Fig. 9, of the probability density function for a diffusion process, *X*_*t*_, through an absorbing boundary, *a*(*t*), which may be time-varying, as the notation suggests. A process *X*_*t*_ = *a*(*t*), starting at zero at time *t* = 0 and located at a point on the boundary at time *t* must have either reached the boundary for the first time at *t* or at some earlier time *τ*, *τ* < *t*. If the latter, then it must have transitioned from the point *a*(*τ*) at time *τ* to the point *a*(*t*) at time *t*, possibly with further boundary crossings in the interval [*τ*, *t*]. Because of the Markov nature of the process, the probability density of the event *X*_*t*_ = *a*(*t*) can be decomposed into the product of two densities: first, the probability that the process makes a first boundary crossing at *τ*; second, the probability that, starting at *a*(*τ*) at time *τ*, it then transitions to state *a*(*t*) at time *t*. Because the event that *X*_*t*_ makes its first boundary crossing at time *τ*, *τ* ∈ (0, *t*), is one of a set of mutually exclusive events, the probability density of the point *X*_*t*_ = *a*(*t*) can be obtained by integrating the product of these two densities over all values of *τ* from 0 to *t*.

To express this symbolically, denote by *f*(*x*, *t*|*y*, *τ*) the free transition density of the diffusion process *X*_*t*_, that is, the probability density that the process, unconstrained by boundaries, starting at *y* at time *τ*, will be found at *x* at time *t*. For the Wiener and OU processes the free transition density is Gaussian but other assumptions about the drift and diffusion rates lead to non-Gaussian distributions. Denote by *g*[*a*(*t*), *t*|*z*, 0] the first-passage time density of the process through the boundary *a*(*t*) at time *t*. The conditional notation expresses the idea that the process transitions from a point *z* at time 0 to the point *a*(*t*) at time *t* and moreover, that this is the first boundary crossing. Fortet’s decomposition states that

A1 $$ f[a(t), t| z, 0] = {{\int}_{0}^{t}} g[a(\tau), \tau |  z, 0] f[a(t), t| a(\tau), \tau] d\tau. $$

The term on the left-hand side is the free transition density of the process starting at *X*_0_ = *z* evaluated at *X*_*t*_ = *a*(*t*). The first term under the integral on the right is the first-passage time density of the process starting at *z* at time 0 through the boundary *a*(*τ*) at *τ*. The second term is the free transition density of the process starting from the boundary point *a*(*τ*) at *τ* and transitioning to a point on the boundary *a*(*t*) at time *t*. This latter point may either be the first boundary crossing or there may be one or more additional crossings before this point. The decomposition in Eq. A1 covers both eventualities.

Equation A1 is a Volterra integral equation of the first kind, in which the unknown function of interest, the first-passage time density function *g*[*a*(*t*), *t*|*z*, 0], appears under the integral sign. For processes with two absorbing boundaries, as often arise in modeling decision processes, there are two such equations, one for each boundary, and the decomposition based on first boundary crossings needs to keep track of crossings at each of them separately (Buonocore et al., 1990; Smith, 2000; Smith and Ratcliff, 2022), but I restrict myself here to the single boundary case, which is the one that arises in models with racing processes. Buonocore, Nobile, and Ricciardi (1987) showed that it is possible to transform Eq. A1 into a Volterra integral equation of the second kind, in which the unknown function appears on the left-hand side and is expressed as the integral of the product of its values at earlier times, *τ* < *t*, and a kernel function, Ψ[*a*(*t*), *t*|*a*(*τ*), *τ*], and showed how it is possible to derive the kernel function for a number of well-known diffusion processes, including the Wiener process and the OU process. When *X*_0_ < *a*(0), that is, the process starts below the boundary, the resulting equation is

A2 $$ \begin{array}{@{}rcl@{}} g[a(t), t| z, 0] &=& -2{\Psi}[a(t), t | z, 0] \\&&+ 2 {{\int}_{0}^{t}} g[a(\tau), \tau |  z, 0] {\Psi}[a(t), t| a(\tau), \tau] d\tau, \end{array} $$

which is Eq. 3 in the text. Equation A2 is obtained from Eq. A1 integrating both sides of the equation over the state variable *x* from *a*(*t*) to $\infty $, then by exchanging the order of integration, then by differentiating the equation with respect to time, and finally by considering the limit of the resulting double integral as $\tau \rightarrow t$. The reader is referred to Smith (2000) or the original article of Buonocore et al., (1987) for details.

Equation A2 is evaluated numerically by discretizing it and evaluating it on the grid *k*Δ, *k* = 1, 2,… by trapezoidal integration. The discretized form of the equation (Buonocore et al., 1990; Smith, 2000, pp. 440–441) is

A3 $$ \begin{array}{@{}rcl@{}} g[a({\Delta}), {\Delta} | z, 0] & = & -2{\Psi}[a({\Delta}), {\Delta} | z, 0]; \\ g[a(k{\Delta}), k{\Delta} | z, 0] & = & -2{\Psi}[a(k{\Delta}), k{\Delta} | z, 0] \\ & &+ 2{\Delta}\sum\limits_{j=1}^{k-1} g[a(j{\Delta}), j{\Delta} | z, 0], \\&&{\Psi}[a(k{\Delta}), k{\Delta} | a(j{\Delta}), j{\Delta}]; k = 2, 3, {\ldots} \end{array} $$

Buonocore et al., (1987) showed that, when the kernel is chosen in the manner they prescribed, the discretized first-passage time density obtained from Eq. A3 converges to the true first-passage time density as Δ becomes small.

## Footnotes

1 Usher and McClelland (2001) called the corresponding constraint in their leaky competing accumulator model “truncation,” which is consistent with their formulation of the model using a discrete-time approximation to a continuous process. The diffusion limit of truncation is reflection.

2 The forward equation is known in physics as the Fokker-Planck equation.

3 The boundary behavior of the LDLIV process is actually a little subtler than this and depends on its parameters (Feller, 1951; Giorno, Nobile, Ricciardi, & Sacerdote, 1989b). When *q* ≤ 0, that is, the stimulus-dependent part of the drift rate is negative, the boundary is an *exit* boundary: Once a process attains such a boundary it exits the space, so it acts like an absorbing boundary. For 0 < *q* < *σ*, zero is a regular boundary, while for *q* ≥ *σ*, zero is an *entrance* boundary: A process can enter the space at such a boundary but never attain it subsequently.

4 I have corrected a typographical error in the expression for the kernel function in Giorno et al., (1989a) (a missing “*e*” denoting exponentiation in the numerator of the third term on the right-hand side in their Eq. 6.4.) I have followed their notation for the LDLIV and reflecting Wiener processes, with two changes to ease the exposition for an audience of decision modelers. As is customary in this area, I denote the absorbing boundary as *a*(*t*), whereas they denote it *S*(*t*). They use the symbol *r* to denote both the reflecting boundary for the Wiener process and the scaling constant for the diffusion coefficient for the LDLIV model. To avoid the notational clash I retain *r* to denote the reflecting boundary for the Wiener process, but use *σ* to denote the scaling constant for the LDLIV process.

5 Giorno et al., (1989a) write the last term term of Eq. 7 in terms of the Gaussian error function, Erf(⋅), rather than the normal distribution function, Φ(⋅). The two are related by $1 - \text {Erf}(x) = 2{\Phi }(-\sqrt {2}x)$.

6 In a recent survey of the understanding of the central limit theorem by researchers in psychology, Zhang, Olvera Astivia, Kroc, and Zumbo (2022) emphasized that finiteness of the first and second moments is needed for convergence to a normal probability law and argued that this aspect of the theorem is not well understood by researchers in the field. Zhang et al.’s point is almost but not quite correct. The classical central limit theorem is one of a number of results in the theory of stable distributions (Breiman, 1968) that characterize the possible forms of the limiting distribution obtainable from a scaled sum of random variables. Breiman points out that the domain of attraction of the normal probability law contains distributions with finite second moments but is not restricted to them, so finiteness of the second moment is a sufficient but not necessary condition. The necessary condition, which is abstract and technical (Breiman, 1968, p. 214, Eq. 9.42), was given by Gnedenko and Kolmogorov (1954).

## Code Availability

Code for fitting the models described in this study is publicly available at https://osf.io/8na3f.
